# HIV-1 DNA-capture-seq is a useful tool for the comprehensive characterization of HIV-1 provirus

**DOI:** 10.1038/s41598-019-48681-5

**Published:** 2019-08-23

**Authors:** Saori C. Iwase, Paola Miyazato, Hiroo Katsuya, Saiful Islam, Benjy Tan Jek Yang, Jumpei Ito, Misaki Matsuo, Hiroaki Takeuchi, Takaomi Ishida, Kouki Matsuda, Kenji Maeda, Yorifumi Satou

**Affiliations:** 1 Division of Genomics and Transcriptomics, Joint Research Center for Human Retrovirus Infection, Kumamoto University, Kumamoto, Japan; 2 International Research Center for Medical Sciences (IRCMS), Kumamoto University, Kumamoto, Japan; 3 Laboratory of Systems Virology, Institute for Frontier Life and Medical Sciences, Kyoto University, Kyoto, Japan; 4 Division of Human Genetics, Department of Integrated Genetics, National Institute of Genetics, Shizuoka, Japan; 5 Department of Molecular Virology, Tokyo Medical and Dental University, Tokyo, Japan; 6 China-Japan Joint Laboratory of Molecular Immunology & Microbiology, Institute of Microbiology, Chinese Academy of Sciences, Beijing, P.R. China; 7 Research Center for Asian Infectious Diseases, The Institute of Medical Science, The University of Tokyo, Tokyo, Japan; 8 National Center for Global Health and Medicine Research Institute, Tokyo, Japan

**Keywords:** Pathogens, Retrovirus

## Abstract

Regardless of recent advances in the development of anti-retroviral drugs, it is still extremely difficult to eradicate HIV-1 from infected individuals. The characterization of the HIV-1 provirus, a type of viral reservoir, with a high resolution is key to HIV-1 cure research. Here, we demonstrate that DNA-capture-seq is a powerful tool to obtain comprehensive information on the HIV-1 provirus. We use biotinylated DNA probes targeting the entire HIV-1 sequence to capture fragments containing HIV-1 sequences from DNA-seq libraries prepared for high throughput sequencing. We demonstrate that the protocol provided the entire proviral sequence from the beginning of the 5′ LTR to the end of the 3′ LTR. Since HIV-1 DNA-probes can hybridize not only viral fragments but also virus-host chimeric ones, the viral integration site information can also be obtained. We verify the efficiency of the protocol by using latently infected cell lines, such as ACH-2 and J1.1, and newly generated ones. The results reveal that the 2 new clones that we analyse harbour one copy of replication-competent provirus, suggesting that latency is not caused by genetic mutations or deletions of the provirus. In conclusion, HIV-1 DNA-capture-seq is a powerful tool to characterize the HIV-1 provirus at a single nucleotide resolution and therefore might be useful for various experiments aiming for an HIV-1 cure.

## Introduction

Human immunodeficiency virus type 1 (HIV-1) is an exogenous retrovirus that causes acquired immunodeficiency syndrome (AIDS). Since its discovery in the early 1980’s^1,2^, it has infected more than 70 million people globally (WHO, https://www.who.int/gho/hiv/en/). As a part of the retroviral life cycle, the viral RNA genome is reverse-transcribed into double stranded DNA, which is integrated into the host genomic DNA^3,4^. The integrated virus, called a provirus, serves as template to produce new virions that spread the infection to un-infected cells. Extensive research led to the discovery of several drugs that target the virus at different stages in its life cycle, resulting in the establishment of a combined antiretroviral therapy (cART) regime that has made it possible to prevent or delay the development of disease^5,6^. In individuals receiving cART, the plasma HIV-1 RNA dramatically decreases to undetectable levels^7,8^. However, the presence of HIV-1 as an integrated provirus in the infected cells allows the virus to avoid not only the effects of the anti-retroviral drugs but also the host immune surveillance, making it extremely difficult to eradicate the virus completely. Consequently, even if the incidence of infection decreases, the prevalence has increased because people under cART live longer^9^. Therapy cannot be interrupted or suspended due to the possibility of viral rebound and accelerated HIV-related disease progression; thus, this treatment constitutes a psychological burden for the infected individuals in that they must undergo treatment for life^10^.

HIV cure is therefore an important topic in current medical sciences. The HIV-1 reservoir *in vivo* is shaped by multiple factors, including tissue reservoirs, antiviral host immunity, heterogenous infected cell clones and viral sequence, such as variation, mutation and deletion. It has been reported that more than 90% of HIV-1 proviruses in peripheral blood mononuclear cells (PBMCs) are defective^11^. Although defective proviruses might still play a role in keeping an active anti-HIV-1 immune response in infected patients^12^, replication-competent intact proviruses should be targeted to achieve an HIV cure *in vivo*. Recent studies have revealed how the HIV-1 reservoir is maintained *in vivo* in patients^13,14^. When we think about a strategy to eradicate the HIV reservoir *in vivo*, such as the “Shock and Kill” strategy, the first approach would be to evaluate drugs for treatment *in vitro* in latent model cell lines such as ACH-2^15,16^, J1.1^17^, U1^18^, and J-Lat cells^19^. Furthermore, new model cell lines are constantly being generated, such as an HIV-1 provirus with a luciferase reporter to monitor proviral expression in a highly sensitive manner^20^. To understand the mechanism of HIV-1 latency and latency-reversal in these cell models, it is essential to characterize the provirus in these cell lines, including the sequence from the beginning of the 5′LTR to the end of the 3′LTR, its structure and the integration site within the human genome.

DNA sequencing technologies have made impressive advances in the last few years. High-throughput sequencing has been used to characterize HIV-1 proviruses with increasing resolution and accuracy^21–24^. Several experimental methods have been established to analyse different aspects of the proviral sequence. Among these, ligation-mediated-PCR (LM-PCR) is used to detect integration sites with high sensitivity^25–28^, and single-genome PCR allows the assessment of the integrity of the provirus, in addition to its sequence, by using virus-specific primers^14,21,29^. More recently, probe-based capture approaches have further increased the sensitivity and specificity of integration site analysis^30^. We have also previously reported the use of capture probes for the genetic and epigenetic analyses of integrated retroviruses^31^. We later developed an improved analytical approach for this method that allows not only the determination of the entire proviral sequence but also the identification of the integration site with the same dataset using samples that were obtained from HTLV-1-infected patients (Katsuya H, Islam S, *et al*., doi.org/10.2139/ssrn.3354888). Here, we have used this novel method to demonstrate that it can be applied for commonly used HIV-1-infected cell lines, in addition to newly established ones, for their characterization and reliable use in this research field.

## Results

### Efficiency of HIV-1 DNA-capture-seq analysis in latently infected cell lines

**Figure 1:**
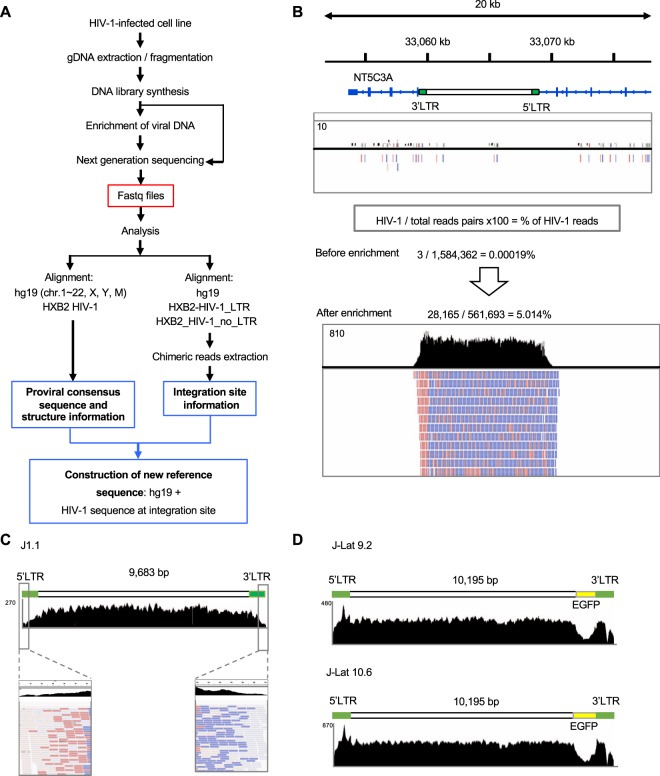
DNA-capture-seq enables the detection of proviral DNA with high sensitivity and the determination of the retroviral integration site. (**A**) The schematic flow of the experimental procedure following the construction of a new reference genome to be used in the analysis of a novel HIV-1-infected cell line. The alignment to a reference sequence, which was comprised of the human genome that includes the HXB2 HIV-1 sequence as an additional chromosome, allows us to obtain the consensus sequence of the integrated provirus and determine its structure. By using the same dataset to align to hg19 and the proviral sequence separated in two (LTR sequence and non-LTR sequence) we obtain the information of the integration site. With this information, we are able to construct a new reference sequence that is specific for the new cell line. (**B**) IGV profile for ACH-2 cell line, before (top) and after (bottom) enrichment with the virus-specific DNA probes. The proportion of reads that aligned to the provirus within the total data is also shown. As a reference genome for both mapping and visualization, we used hg19 containing the HIV-1 proviral sequence inserted at the viral integration site determined for ACH-2 cells. The pink and purple lines depict individual reads aligning the sense and anti-sense strands of the genomic DNA, respectively. (**C**) IGV profile for the J1.1 cell line after enrichment. The initial part of the 5′LTR and the end of the 3′LTR are shown with the details of the reads in the enlarged frames. (**D**) IGV profiles for J-Lat 9.2 and J-Lat 10.6 cell lines. The coverage for the EGFP region is low compared to the rest of the provirus because probes targeting this sequence were not included in the enrichment.

We previously reported that DNA-capture sequencing for retroviruses, HIV-1 and HTLV-1 was useful for detecting retroviral sequences with high sensitivity^31^. To extend the application of the method, we aimed to establish an analytic protocol to perform a more comprehensive analysis of the HIV-1 provirus that would provide information on the entire proviral sequence, its structure, and the viral integration site. We analysed a latently infected cell line, ACH-2, following the experimental workflow shown in Fig. 1A. We extracted gDNA from the cells, fragmented it by sonication, and then prepared the DNA-seq libraries. An aliquot of the library was directly sequenced. We analysed the data using a standard alignment software, bwa^32^, and a reference genome consisting of the human genome (hg19) and HIV-1 genome (HXB2) as an additional chromosome. From a total of approximately 1.6 × 10^6^ reads, only 3 aligned with the provirus (Fig. 1B). To increase the efficiency of viral sequence detection, we performed DNA-capture enrichment with DNA probes covering the entire HIV-1 proviral sequence^31^ before proceeding to the sequencing step. In this case, we obtained more than 28,000 reads aligning with the provirus from a total of over 560,000 mapped reads, demonstrating that HIV-1 DNA-capture markedly increased the sensitivity of viral sequence detection by over 25,000 times (Fig. 1B). We also performed this analysis using J1.1 (Fig. 1C), J-Lat 9.2 and J-Lat 10.6 DNA (Fig. 1D). We were able to obtain the entire proviral sequence in these cell lines, from the beginning of the 5′LTR to the end of the 3′LTR (Fig. 1C), which cannot be achieved by conventional deep sequencing protocols using virus-specific primers. Both the 5′ and the 3′LTR of HIV-1 are generally identical sequences; therefore, we cannot distinguish them based on their DNA sequences. Consistent with the fact that there were no probes for GFP, we observed low sequencing coverage in the GFP region of the J-Lat 9.2 and J-Lat 10.6 cells^19^ (Fig. 1D). Since the virus-enriched sample showed relatively even coverage along the entire length of the proviral sequence (Fig. 1B–D), we can say that the probes could enrich each proviral region with similar efficiencies.

### Integration site analysis by HIV-1 DNA-capture-seq

**Figure 2:**
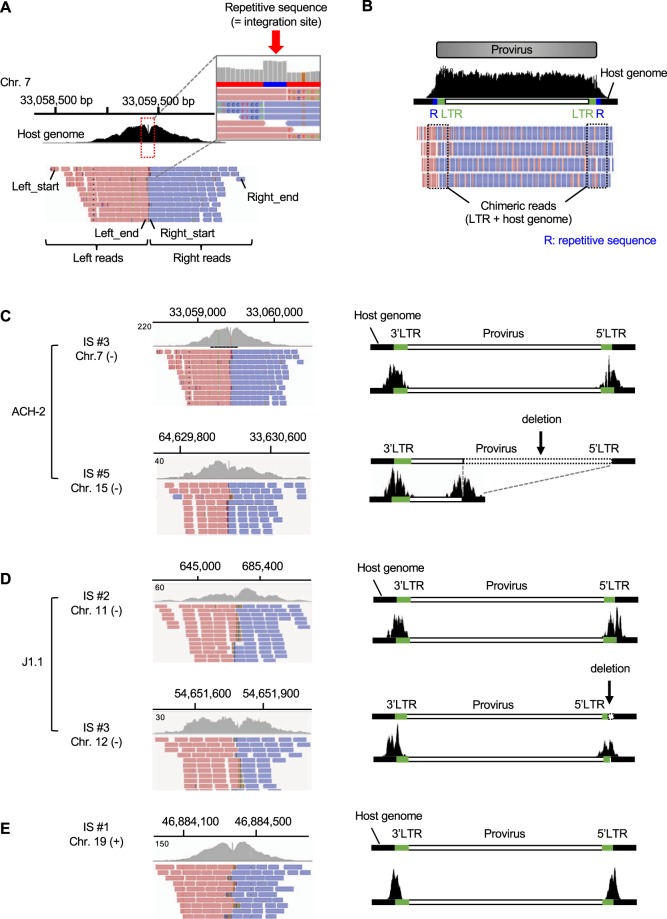
Integration sites can be determined with the DNA-capture-seq datasets. (**A**) The IGV profile of virus-host chimeric reads in the ACH-2 dataset, mapped to the hg19 reference sequence alone. The human portion of the chimeric reads can be observed together with the 5-nucleotide long repetitive sequence (R) that results from the retroviral integration step at the integration site. (**B**) The IGV profile of the integrated provirus after alignment to the newly constructed reference sequence. Since the proviral sequence is integrated within the corresponding chromosome in the reference sequence used for alignment, the reads in the IGV profile show the chimeric fragments, including the junctions. (**C**) The IGV profiles of the human fragments of virus-host chimeric reads obtained from the ACH-2 cell line’s dataset at the integration site (IS) (left). Two major proviruses were shown, and one was a defective provirus. In this case, both clones were found to be integrated in the minus strand of the genome (−). The localization of the chimeric reads along the reference sequence containing the integrated provirus is shown on the right. The same information is shown for J1.1 (**D**) and J-Lat 9.2 clone (**E**).

Each DNA probe that was used in the HIV-1 DNA-capture seq is 120 bp long. Most of the reads in the DNA-seq libraries that were prepared in this study ranged from 300 to 500 bp in length. Virus-host chimeric fragments containing the junction between the viral LTR and the flanking genomic DNA could be captured by the DNA probes. Thus, it would be possible to obtain the information of the sequence next to the HIV-1 provirus and thereby determine the integration site. To test whether we could analyse HIV-1 integration site with the DNA-seq data, we re-analysed the datasets that we obtained in Fig. 1. Based on the average length of the DNA-seq reads, which ranged from 300 to 500 bp, the virus-host chimeric reads generally contain the HIV-1 LTR sequence because these are present at both ends of the provirus and are 634-bp in length. Since the 5′LTR and 3′LTR sequences are identical, we cannot be certain about the origin of the reads that are aligned with the proviral LTRs. We therefore used a custom-made hg19 reference genome with HIV-1 LTR and HIV-1 without LTR (HIV-1 noLTR) as additional chromosomes and made a Perl program to extract the virus-host chimeric reads from the aligned data. The chimeric reads that were obtained from the ACH-2 cell line were visualized on IGV (Fig. 2A). As reported previously, HIV-1 integration site was located at position 33,059,403–33,059,404 in chromosome 7^33^. There was a significant accumulation of chimeric reads around that reported integration site. Since there is no HIV-1 sequence in chromosome 7 in the reference genome that was used for the mapping, only the host sequences of the chimeric reads were mapped to the region (Fig. 2A). Retroviruses generally introduce sequence replicates at their integration site when they integrate into the host genome using the viral integrase^34^. In the case of HIV-1, the virus generates a 5-bp repeat at both ends of integrated provirus, resulting in a 5-bp repeat next to the provirus (Fig. 2A). Based on the consensus sequence and the integration site information that we obtained from the ACH-2 cells, we generated a custom genome for this cell line, in which the HIV-1 sequence and the 5-bp repeat were inserted in the integration site. Next, we performed the mapping step again, this time to the custom-made reference genome, and visualized the mapping result on IGV (Fig. 2B). A list of the chimeric reads that were detected in the analysis of the ACH-2 cells is shown in Table 1. As reported previously, there was an additional integration site in chr15^33^. We also identified the same integration site in this study, and the mapping result was visualized on IGV (Fig. 2C). Based on the mapping position of the viral reads in the HIV-1 provirus, we found that HIV-1 was integrated in a direction opposite to the host genome and that the provirus was defective (Fig. 2C, lower panel). There were various chimeric reads in addition to the two integration sites that we analysed (Table 1), indicating that there might be experimental artefacts generated during the DNA library preparation process (Fig. 1A). In particular, the ligation of fragmented DNA is likely to experimentally generate virus-host chimeric reads. To test this possibility, we analysed a negative control DNA sample that was made by mixing the DNA of Jurkat T cells and an HIV-1 plasmid, NL4-3^35^, in which there is no integration of HIV-1 into the host genome. We analysed the negative control with the same sequencing depth as the ACH-2 cell line and found that there were no chimeric reads similar to IS #3 and #5 in ACH-2 cells (Supplementary Table S1 and Table 1). Based on these results, we defined HIV-1 integration site as a region containing two pairs of virus-host chimeric reads, where the host sequences at both sides of the provirus are convergent and within a distance of 500 bp. The minor integration sites, such as IS #1, #2 and #4 (Table 1) should be generated during long-term cultivation after clone establishment. We further analysed the data that we obtained from J1.1 cell line and identified HIV-1 integration site by using the criteria defined above. Same as in the case of ACH-2 cells, we found that the J1.1 cells harboured two major proviruses (Table 2); one was a full-length type, and the other was defective in the 5′LTR (Fig. 2D), showing that HIV-1 DNA-seq efficiently detected the structural abnormalities of the HIV-1 proviruses. We also analysed the J-Lat 9.2 cells and found that they contained one copy of a full-length type provirus in the host cellular genome (Fig. 2E and Table 3). These results collectively indicate that the HIV-1 DNA-capture-seq is useful to analyse HIV-1 integration site in a comprehensive manner.

**Table 1:** A list of the chimeric reads detected in the analysis of ACH-2 cells (Top 10 reads are shown).

IS	Left reads of IS				Right reads of IS				Total reads	Strand
	Chr	Start site	End site	# reads	Chr	Start site	End site	# reads		
#1	chr1	121484766	121484813	1	chr1	121485140	121485216	1	2	−
#2	chr3	106552333	106552548	2	chr3	106552684	106552760	1	3	−
#3	chr7	33058453	33059404	723	chr7	33059397	33060344	695	1418	−
#4	chr15	52449478	52449554	1	chr15	52449692	52449937	5	6	−
#5	chr15	64629463	64630237	85	chr15	64630232	64630902	77	162	−
#6	chr1	17848557	17848633	1	.	.	.	.	1	−
#7	chr1	19236464	19236540	1	.	.	.	.	1	−
#8	chr1	46736503	46736579	1	.	.	.	.	1	−
#9	chr1	55243102	55243178	1	.	.	.	.	1	+
#10	chr1	55561350	55561426	2	.	.	.	.	2	−

**Table 2:** A list of the chimeric reads detected in the analysis of J1.1 cell (Top 10 reads are shown).

IS	Left read of IS				Right reads of IS				Total reads	Strand
	Chr	Start site	End site	# reads	Chr	Start site	End site	# reads		
#1	chr3	49815218	49815293	1	chr3	49815620	49815696	1	2	−
#2	chr11	684719	685244	84	chr11	685238	685721	97	181	−
#3	chr12	54651116	54651758	75	chr12	54651752	54652181	47	122	−
#4	chr1	4925435	4925511	1	.	.	.	.	1	−
#5	chr1	32116270	32116346	1	.	.	.	.	1	+
#6	chr1	59477002	59477077	1	.	.	.	.	1	−
#7	chr1	68686233	68686308	1	.	.	.	.	1	+
#8	chr1	98403969	98404045	1	.	.	.	.	1	−
#9	chr1	101661625	101661701	1	.	.	.	.	1	+
#10	chr1	115758154	115758230	1	.	.	.	.	1	−

**Table 3:** A list of the chimeric reads detected in the analysis of J-Lat 9.2 (Top 10 reads are shown).

IS	Left read of IS				Right reads of IS				Total reads	Strand
	Chr	Start site	End site	# reads	Chr	Start site	End site	# reads		
#1	chr9	139362636	139363037	390	chr9	139363030	139363527	430	820	+
#2	chr1	52076930	52077027	1	.	.	.	.	1	−
#3	chr1	102847012	102847067	1	.	.	.	.	1	−
#4	chr1	158229656	158229711	1	.	.	.	.	1	+
#5	chr1	196007120	196007175	1	.	.	.	.	1	+
#6	chr1	198911734	198911831	1	.	.	.	.	1	−
#7	chr1	205637421	205637476	1	.	.	.	.	1	+
#8	chr2	154142985	154143040	1	.	.	.	.	1	+
#9	chr2	160762841	160762896	1	.	.	.	.	1	+
#10	chr2	161255676	161255731	1	.	.	.	.	1	−

### Application of DNA-capture-seq for newly established HIV-1-infected clones

**Figure 3:**
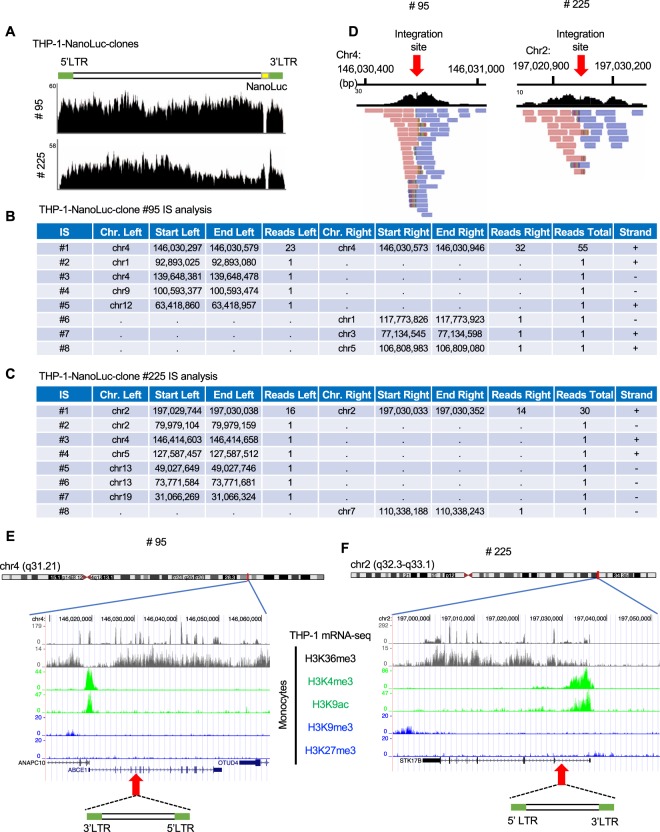
Characterization of newly established clones by DNA-capture-seq. (**A**) IGV profiles showing the complete proviral sequence of THP-1-NanoLuc clones #95 and #225. The integration sites detected after the analyses are shown for clone # 95 (**B**) and clone #225 (**C**). In both cases, only one integration site (IS) fulfilled the conditions that were set for the determination of a valid integration site (IS #1 for both clones). (**D**) The IGV profile of the human portion of the virus-host chimeric reads mapped to the hg19 reference sequence alone are shown. The genetic and epigenetic environments of the integrated proviruses in clones #95 (**E**) and #225 (**F**) are shown. RNA-seq data of the THP-1 cells and ChIP-seq datasets of CD14^+^ monocytes were obtained from the NCBI Sequence Read Archive (SRA) database and UCSC Genome Browser, respectively.

New models of HIV-1 latent infection are being developed for HIV cure research. We recently established an *in vitro* model of HIV-1 latent infection with a monocyte-derived cell line, THP-1. The THP-1 cells were infected with an HIV-1 molecular clone containing the NanoLuc reporter to monitor proviral expression with high sensitivity^20^. We found two clones, #95 and #225, with low basal levels of proviral expression that could be reactivated upon stimulation by latency-reversing agents, such as SAHA and JQ-1^20^. Based on these results, we thought that the established cell lines would be useful for the analysis of HIV-1 latency, but the proviruses in the clones have not been characterized yet. We therefore performed an HIV-1 DNA-capture-seq analysis to obtain the entire proviral sequence and to determine its structure and integration site. First, we analysed the proviral sequence and found that it perfectly matched the sequence of the molecular clone that was used for infection, demonstrating that mutations and/or deletions in the provirus are not the cause of the latency of the clones (Fig. 3A). Note that the sequence along the *NanoLuc* gene showed low sequencing coverage, since the probes that we designed do not cover it, and it was not sufficiently enriched. Next, to determine the integration site of each clone, we extracted virus-host chimeric reads and aligned them to the hg19 reference genome. The generated lists of integration sites confirmed that there was only one clone expanding in each THP-1 clone (Fig. 3B,C). Convergent reads corresponding to the human genome fragments of chimeric reads were observed around the integration site when they were visualized in IGV (Fig. 3D). The presence of the 5-bp-repeat sequences in both clones (#95 and #225) indicated that HIV-1 integrated using its integrase enzyme in both cases. We then looked at the genomic environment at the integration site in these clones and found that the HIV-1 provirus was integrated into the *ABCE1* and *STK17B* genes in clones # 95 and # 225, respectively. Based on the RNA-seq and ChIP-seq datasets that we found in the public databases for THP-1 cells and CD14^+^ monocytes, HIV-1 proviruses were integrated into transcriptionally active and open chromatin regions (Fig. 3E,F). These findings demonstrated that HIV-1 DNA-capture-seq is a powerful tool to perform a comprehensive characterization of HIV-1 proviruses in newly established HIV-1-infected clones and cell lines.

### HIV-1 DNA-capture-seq analysis for a heterogeneous population of infected clones

**Figure 4:**
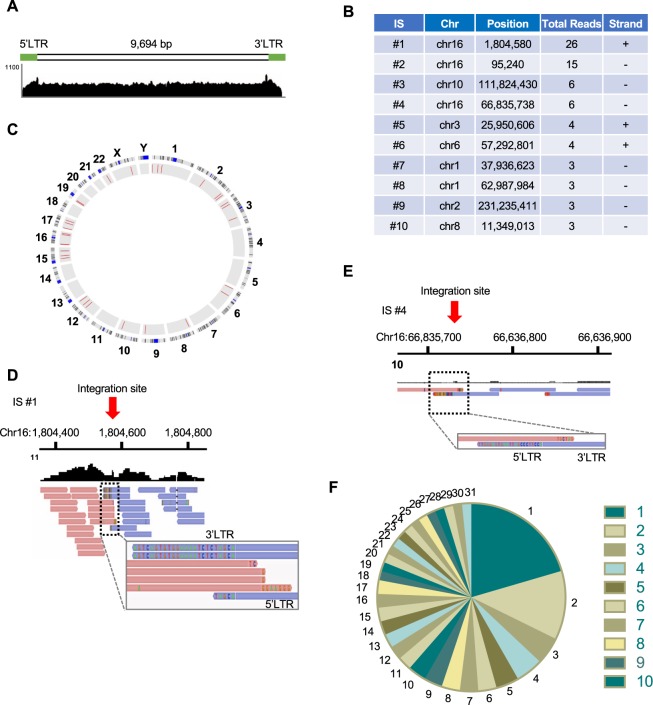
Characterization by DNA-capture-seq of HIV-1 provirus in Jurkat cells infected with NL4-3, an HIV-1 infectious clone. (**A**) The IGV profile showing the cumulative sequence coverage that was obtained from the infected cells analysed in bulk. (**B**) Table of some of the integration sites that were detected in the infected cells. (**C**) Circos plot showing each chromosome in the outer circle, and the identified integration sites are shown as red lines in the inner circle. The details of integration site ID #1 (**D**) and #4 (**E**) are shown, as visualized with IGV. The presence of overlapping sense and anti-sense sequences that include the repetitive sequence at the integration site was observed. (**F**) Pie chart showing the relative abundance of each of the infected clones that were detected by the DNA-capture-seq in Jurkat cells infected with NL4-3. The numbers represent each of the infected clone in the integration site analysis.

We demonstrated the usefulness of HIV-1 DNA-capture-seq for HIV-1 provirus analysis in cell lines and clones in which there were one or two dominant infected clones in the sample. We next aimed to test the efficiency of the protocol on the characterization of HIV-1 proviruses in a sample harbouring heterogeneous infected clones. For that purpose, we infected Jurkat T cells with the HIV-1 infectious molecular clone NL4-3 and cultured the cells for more than one year. Then, we harvested the cells, extracted gDNA, and performed HIV-1 DNA-capture-seq. For the alignment of the sequencing reads, we used the custom reference genome consisting of the human genome (hg19) and HIV-1 as an additional chromosome. Since there were multiple infected clones in this sample, the cumulative sequence and coverage value from each provirus was obtained and visualized on IGV (Fig. 4A). Next, we analysed the integration sites following the procedure that was established in this study (see Methods for more details). There were 31 unique integration sites identified, and 10 of them are listed in the table in Fig. 4B. The detected HIV-1 integration sites were visualized in a Circos plot, which showed a wide distribution comprising various chromosomes (Fig. 4C). Because of the heterogeneity of the infected clones, the coverage for virus-host chimeric reads was not as high as the results that were obtained when analysing infected cell lines, such as ACH-2 (Fig. 2A). Although there was a small number of reads around the integration sites, the virus-host reads were derived from host-5′LTR and 3′LTR-host junctions. More importantly, the presence of the repeat sequences at both the 5′- and 3′-sides of the HIV-1 provirus was confirmed (Fig. 4D,E), indicating that the chimeric reads were generated from an HIV-1-integrase-mediated integration process. In the ligation-mediated PCR protocol, one can estimate the abundance of retroviral integration sites from the DNA fragment length data^36^. Since we also used sonication to fragment DNA for the NGS library synthesis in the DNA-capture-seq, we would be able to use the DNA fragment length information to estimate the relative abundance of the infected clones. We compared clonal abundance that we obtained by either the DNA-capture-seq or digital droplet PCR (ddPCR) targeting for virus-host junction of two major proviruses in ACH-2 cells (Table 1). Both the DNA-capture-seq and ddPCR analyses showed similar values of clonal abundance for ACH-2 #3 and #5 (Supplementary Fig. S1A), indicating that DNA-capture-seq provides information about the relative abundance of each proviruses in the sample. We analysed the DNA from the Jurkat/NL4-3 cells and found that some clones were expanded (Fig. 4F). These results indicate that the HIV-1 DNA-capture-seq method would also be useful for the characterization of the HIV-1 provirus in samples containing heterogeneous infected clones, especially for integration site and clonality analysis of infected cells.

## Discussion

HIV cure research is currently one of the hottest fields in medical sciences. A main obstacle for HIV cure is the presence of the integrated HIV-1 proviruses in the host cellular genomic DNA because the host immune system and current anti-retroviral drugs cannot target them. To elucidate the underlying mechanisms for HIV-1 latency, the demand for more precise and accurate evaluation methods of HIV-1 provirus is increasing every year. Thus far, various protocols have been developed to characterize HIV-1 proviruses, such as deep sequencing using virus-specific primers, ligation-mediated PCR to characterize viral integration sites^27,28^, the full-length individual proviral sequencing (FLIPS) assay^21^, and matched integration site and proviral sequencing (MIP-Seq)^29^. In addition to these, we have shown in this study that DNA-capture-seq is a useful protocol to characterize the HIV-1 provirus. Each protocol has both advantages and disadvantages in terms of obtainable proviral information, throughput, required cost, time, and expertise. The DNA-capture-seq protocol provides comprehensive information in a single assay, including whole proviral sequence, structure of the provirus, and viral integration site. The experimental flow does not contain a step for a DNA-limiting dilution to obtain just one HIV-1 genome in each reaction. This makes the protocol much simpler than other assays aiming to amplify single genomes, such as FLIPS assay or MIP-Seq, but one cannot obtain information on the individual proviral sequence with DNA-capture-seq. As demonstrated in this study, DNA-capture-seq will be especially useful in samples in which there is less heterogeneity of the clones, such as *in vitro* infected clones or *in vitro* or *in vivo* models using molecular clones (Supplementary Table S2). We also evaluated detection sensitivity of the viral integration site using ACH-2 cells. There are two dominant proviruses in ACH-2 cells, ACH-2 #3 and ACH-2 #5 (Table 1). The ddPCR quantification revealed that the proportions of ACH-2 #3 and ACH-2 #5 were 85.9% and 14.1%, respectively. A total of 1,000 reads were enough to detect ACH-2 #3, whereas 5,000 reads were required for ACH-2 #5 (Supplementary Fig. S1B), indicating that the expanded clones are detectable by the DNA-capture-seq but that a large number of sequencing reads would be required to detect the non-expanded clones that we normally observe in HIV-1-infected individuals.

Generally, a nucleic acid hybridization protocol is sensitive to sequence mismatches. We have previously demonstrated that eight or fewer mismatches in 120 bp probes do not affect the target-capture efficiency^31^. However, HIV-1 infection generally contains a high frequency of mutations and deletions in the provirus. We further investigated how sequence mismatches affect the efficiency of the HIV-DNA-capture protocol using U1 cells. These cells present a wider range of mutations when compared to the HXB2-based probes (Supplementary Fig. S2). The results showed the tendency that a higher degree of mismatches per probe decreased the enrichment efficiency, although there was no statistical significance. One alternative to overcome this problem would be adding more DNA-probes to cover the heterogeneous viral sequences, as shown in a previous study aiming to capture a wide range of HIV-1 LTR sequences and showing the ability to analyse HIV-1 integration site in patients^30^. Another hurdle when analysing clinical samples is the very low frequency of infected cells in the PBMCs of HIV-1-infected individuals, where the proviral load is generally below 0.1%. Further optimization of the protocol, such as a second-round probe-enrichment, will be required to apply this protocol to analysing clinical materials.

Taken together, one needs to select an optimal experimental protocol to analyse HIV-1 proviruses by considering the priority of the information for each study. HIV-1 DNA-capture-seq would be suitable for a comprehensive proviral analysis of infected cell populations with relatively low heterogeneity in a high-throughput manner. A phylogenetic analysis of the HIV-1 sequence would also need some modifications to the current analytical pipeline. The proviral sequences that were obtained by this analysis are based on the cumulative coverage provided by the sequencing signal. The detection of more than one sequence within the dataset will require a statistical assessment of minor variants.

It is well-known that defective proviruses emerge from the initial phase of infection in infected individuals^37^. Since defective proviruses generally would not serve as a template for progeny virus production, we do not have to eradicate them in order to aim to achieve an HIV cure. Rather, we need to tackle the replication-competent proviruses that are transcriptionally silenced but can be reactivated by changes in the intracellular and extracellular circumstances^11^. There are multiple layers of regulatory mechanisms for such transient and reversible HIV-1 latency. The human genome is epigenetically well-orchestrated to translate the genetic code that is written in the DNA into various proteins and RNA molecules to exert such biological processes. Although HIV-1 is preferentially integrated into genomic regions with open chromatin^38^, there should be a wide range of genomic and epigenomic microenvironments that are targeted by HIV-1 integration that should be associated with the transcriptional regulation of the integrated provirus. Therefore, not only the viral sequence but also the viral integration site in the host genome is a key factors to understand HIV-1 latency. Taking advantage of HIV-1 DNA-capture-seq, we have characterized latent HIV-1 proviruses in monocyte-derived cell lines (Fig. 3). We found that there were no mutations in the proviruses and that these were integrated into transcriptionally active regions and in the opposite orientation relative to the host genes. It would be interesting to unveil the mechanisms that are involved in the silencing of the proviruses that are integrated in such open chromatin regions. One possible explanation is the transcriptional interference by the host genes. There are two previous papers describing the relationship between HIV-1 latency and the orientation relative to host genes^39,40^. The MIP-Seq demonstrated that there is a higher proportion of intact proviruses integrated in the opposite orientation relative to the host genes in CD4^+^ T cells of HIV-1-infected individuals^29^. Further experiments need to be performed to elucidate how integrated proviruses and the host genes affect each other.

Another key player for HIV-1 latency is epigenetic regulation. Previous studies have demonstrated that DNA methylation^41^ or some types of histone modifications, such as H3K27me3^42^ and H4K20me1^43^, are involved in the silencing of HIV-1 provirus. We have previously reported that DNA-capture-seq increases the detection sensitivity of retroviral sequences^31^. This would be especially useful for ChIP-seq analyses of HIV-1 proviruses, in which the proportion of HIV-1 sequences is extremely low due to the difference in size between the human and retroviral genomes. One would be able to explore the localization of transcription factors and epigenetic modifiers to the HIV-1 provirus and/or characterize the histone modifications on the entire HIV-1 provirus with thousands of times higher sensitivity, as we have shown for HTLV-1-infected cells^44,45^.

In conclusion, we have established a new protocol to characterize HIV-1 proviruses. The protocol provides multiple information in one assay, including the entire proviral sequence, proviral structure, viral integration site, and clonal abundance of infected cells. This new protocol could be useful in furthering various experiments aiming for an HIV-1 cure.

## Methods

### Probes for DNA-capture-seq

HIV-1 subtype B is the most common subtype in Japan, the USA and Europe. In addition, several of the HIV-1-infected cell lines that are most commonly used in research are also of this subtype. Since HXB2 is the reference sequence for this subtype, 161 biotinylated DNA probes (IDT) were custom-designed based on this sequence (GenBank accession number K03455). In the experiment using the NL4-3 molecular clone, additional probes were included in the hybridization mixture to cover viral regions with mismatches between HXB2 and NL4-3 (Supplementary Table S3).

### Cell lines

Several HIV-1-infected latent cell lines were used in the current study. The following cell lines were obtained through the AIDS Research and Reference Reagent Program, Division of AIDS, NIAID, NIH: ACH-2 from Dr. Thomas Folks^16^. The J1.1 cells and U1 cells were obtained from Dr. Thomas Folks^18^, and the J-Lat Full Length GFP cells (clones #9.2 and #10.6) were obtained from Dr. Eric Verdin^19^. The Jurkat cells were obtained from Dr. Arthur Weiss^46^. The THP-1-NanoLuc-clones #95 and #225 had been established previously by infecting the human monocyte-lineage cell line THP-1 with the vesicular stomatitis virus G (VSV-G) pseudotyped NLnNanoLuc-Kp, as previously described^20^. All of the cell lines were cultured in RPMI that was supplemented with 10% heat-inactivated fetal bovine serum, penicillin (100 U/ml) and streptomycin (100 µg/ml). All of the HIV-1-infected cells were handled in bio-containment level 3 rooms.

### Genomic DNA (gDNA) extraction and sample processing

Genomic DNA from the infected cells was extracted using the DNeasy Blood and Tissue kit (Qiagen). Three micrograms of gDNA were sheared by sonication using a Picoruptor (Diagenode) device to an average size of 300 bp. To evaluate the possible detection of false integration sites by our DNA-capture-seq method, a test sample was prepared by mixing 20 µg of Jurkat gDNA with 1 ng of NL4-3 molecular clone in a final volume of 200 μl of EB buffer (Qiagen). This mixture was sheared by sonication using a Bioruptor USD-300 (Diagenode) device to an average size of 300 bp.

### Library preparation

Up to one microgram of gDNA that was processed as described above was used for the synthesis of the libraries using NEBNext Ultra DNA II library preparation kit and NEBNext multiplex oligos for Illumina (New England BioLabs), following the recommendations of the manufacturer. The concentration was determined using a Qubit fluorometer (Thermo Fisher Scientific) before proceeding to the DNA-capture-seq step.

### DNA-capture-seq

For the enrichment of viral fragments contained in the synthesized libraries, several libraries were pooled to perform the capture step, as described before^31^. Briefly, the pooled libraries were mixed with the virus-specific probes in the presence of human Cot-1 DNA (Invitrogen) and xGen universal blocking oligos (IDT) for the hybridization step. A series of wash steps were performed using DNA xGen lockdown reagents (IDT), following the manufacturer’s recommendations. The quality of the enriched DNA libraries was evaluated by electrophoresis with a TapeStation 2200 system (Agilent Technologies) and quantified by real time PCR with the GenNext NGS library quantification kit (Toyobo). Finally, the multiplexed libraries were subjected to cluster generation using a MiSeq Reagent Kit v3 (150 cycles) or NextSeq 500 Kit (75 cycles) in MiSeq or NextSeq desktop sequencing systems (Illumina), respectively.

### Data analysis

The following paired-end sequencing in Illumina devices outputs 3 fastq files were used for the analysis: Read1, Read2 and Index. Paired-reads with Index read’s Phred score > 20 were first selected using an in-house Perl script (kindly provided by Dr. Michi Miura, Imperial College). Subsequently, the adapter sequences were trimmed from the Read1 and Read2 files, and an additional cleaning step was performed by selecting Read1 and Read2 with Phred score > 20. Using these clean reads, alignment to a reference sequence was performed using the BWA-MEM algorithm^32^. To determine the complete sequence of the proviruses in the different cell lines and infer their structure, the reference genome to which they were aligned included the entire human genome (hg19) and the complete HXB2 sequence as an independent chromosome. However, to determine the integration sites, the HXB2 sequence in the reference genome was included as 2 different chromosomes: viral LTRs (HIV_LTR) and proviral sequence without the LTRs (HIV_noLTR). The Samtools program^46,47^ and Picard command line tools (http://broadinstitute.github.io/picard/) were used to remove multiply aligned reads and duplicates, respectively. The THP-1 mRNA-seq and ChIP-seq data were obtained from the NCBI Sequence Read Archive (SRA) database under the accession number SRA458685^48^ and UCSC Genome Browser (http://hgdownload.soe.ucsc.edu/goldenPath/hg19/encodeDCC/); H3K36me3, wgEncodeBroadHistoneMonocd14ro1746H3k36me3Sig.bigWig; H3K4me3, wgEncodeBroadHistoneMonocd14ro1746H3k04me3Sig.bigWig; H3K9ac, wgEncodeBroadHistoneMonocd14ro1746H3k09acSig.bigWig; H3K9me3, wgEncodeBroadHistoneMonocd14ro1746H3k09me3Sig.bigWig; H3K27me3, and wgEncodeBroadHistoneMonocd14ro1746H3k27me3Sig.bigWig. The final aligned files were visualized using the Integrative Genomics Viewer (IGV)^49^.

### Integration site analysis

To determine the integration sites of the proviruses in the different cell lines, alignment was performed as described above, using the reference human genome that included the HXB2 sequence as 2 separate chromosomes (HIV_LTR and HIV_noLTR). From the resulting files, viral-human chimeric reads were extracted using an in-house Python script. Some of these reads are randomly generated during library synthesis (Supplementary Table 1), so only the virus-host chimeric reads fulfilling the following conditions were considered to perform the analysis (Fig. 2A): (1) the human portion of “left reads” and “right reads” have to align to the same chromosome with a convergent orientation; (2) the distinctive 5 bp-repetitive nucleotide sequence generated during the integration process should be present.

### Digital droplet PCR (ddPCR)

Droplet digital PCR (ddPCR) was performed by using primers and a probe targeting a HIV-1 gag region and the *ALB* gene, according to previous reports with minor modifications^50^ or the host-virus junction of major provirus in ACH-2 cells. ddPCR droplets were generated by the QX200 droplet generator (Bio-Rad). Generated droplets were then transferred to a 96-well PCR plate and sealed with a pre-heated PX1^™^ PCR plate sealer (Bio-Rad) for 5 seconds at 180 °C. For probe based ddPCR, PCR were performed with the following settings: 95 °C for 10 minutes followed by 39 cycles of 94 °C for 30 seconds, 58 °C for 60 seconds, and final 98 °C for 10 minutes and 4 °C for hold and for EvaGreen (Bio-Rad) based ddPCR, we followed the following conditions 95 °C for 5 minutes followed by 40 cycles of 95 °C for 30 seconds, 59 °C for 60 seconds, 4 °C for 5 minutes and final 90 °C for 5 minutes and 4 °C for hold. Sequences of primers and probes are shown in Supplementary Table S4. The plate was then placed in the QX200 droplet reader (Bio-Rad) for quantification of the number of positive and negative droplets based on their fluorescence. Threshold values for ddPCR were determined based on the highest level of droplet fluorescence in the no-template-control sample (NTC) to provide an objective cut-off with maximum sensitivity. Data were analyzed using QuantaSoft software (Bio-Rad). Then DNA load was calculated as follows: proviral load (%) = (copy number of HIV-1 gag DNA)/{(copy number of *ALB*)/2} × 100.

### Statistical analysis

The correlation coefficient r was applied to the measurement of the strength and direction of the linear relationship between each probe’s mismatches with U1 provirus and its cumulative sequencing coverage. Statistical analysis was performed by JMP software, version 11 (SAS Institute, Cary, NC).

## Supplementary information

Supplementary information

## Data Availability

The fastq files and bam files that were obtained in this study have been deposited in the NCBI SRA database (accession no. PRJNA524421). Data on the findings reported here are available from the corresponding author upon request.
