# Supplementary material for: HIV-1 DNA-capture-seq is a useful tool for the comprehensive characterization of HIV-1 provirus
Source: Sci Rep. 2019 Aug 23;9:12326. doi: 10.1038/s41598-019-48681-5 (PMC6707141; doi:10.1038/s41598-019-48681-5)
Supplement: Supplementary file 1 — Supplementary information [file 41598_2019_48681_MOESM1_ESM.pdf]

Supplementary information

HIV-1 DNA-capture-seq is a useful tool for the comprehensive characterization of HIV-1 provirus

Saori C. Iwase[1,2], Paola Miyazato[1,2], Hiroo Katsuya[1,2], Saiful Islam[1,2], Benjy Tan Jek Yang[1,2], Jumpei Ito[3,4], Misaki Matsuo[1,2], Hiroaki Takeuchi[5], Takaomi Ishida[6,7], Kouki Matsuda[8], Kenji Maeda[8], Yorifumi Satou[1,2]*

A

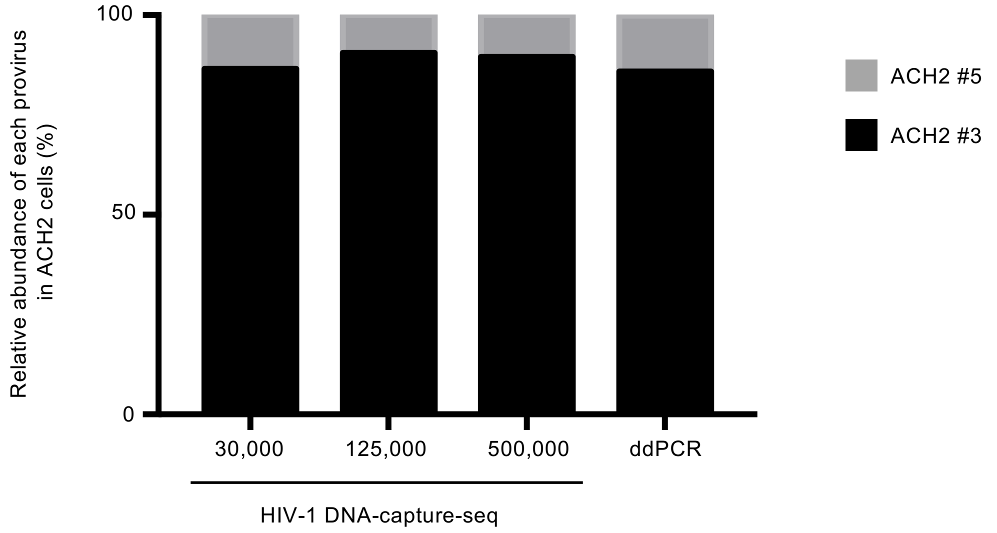

B

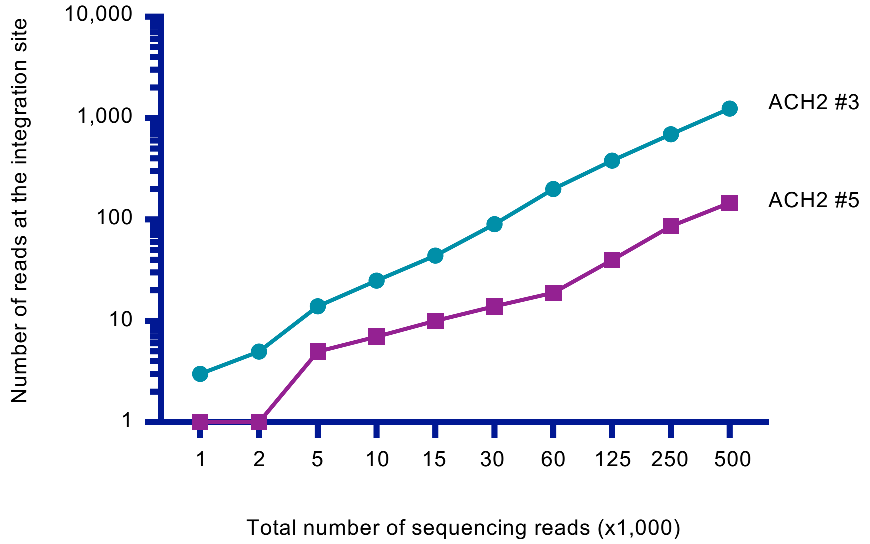

Supplementary Figure S1 HIV-1 DNA-capture-seq using ACH2 cells
A) Relative abundance of each provirus in ACH2 cells (%). Values shown are generated by the DNA-capture-seq of 30,000, 125,000, or 500,000 total reads. The value obtained by digital droplet PCR was also shown as a reference value. ACH2 #3 and #5 are the same ID as Table 1.
B) A relationship between number of reads at the integration site and total number of sequencing reads.

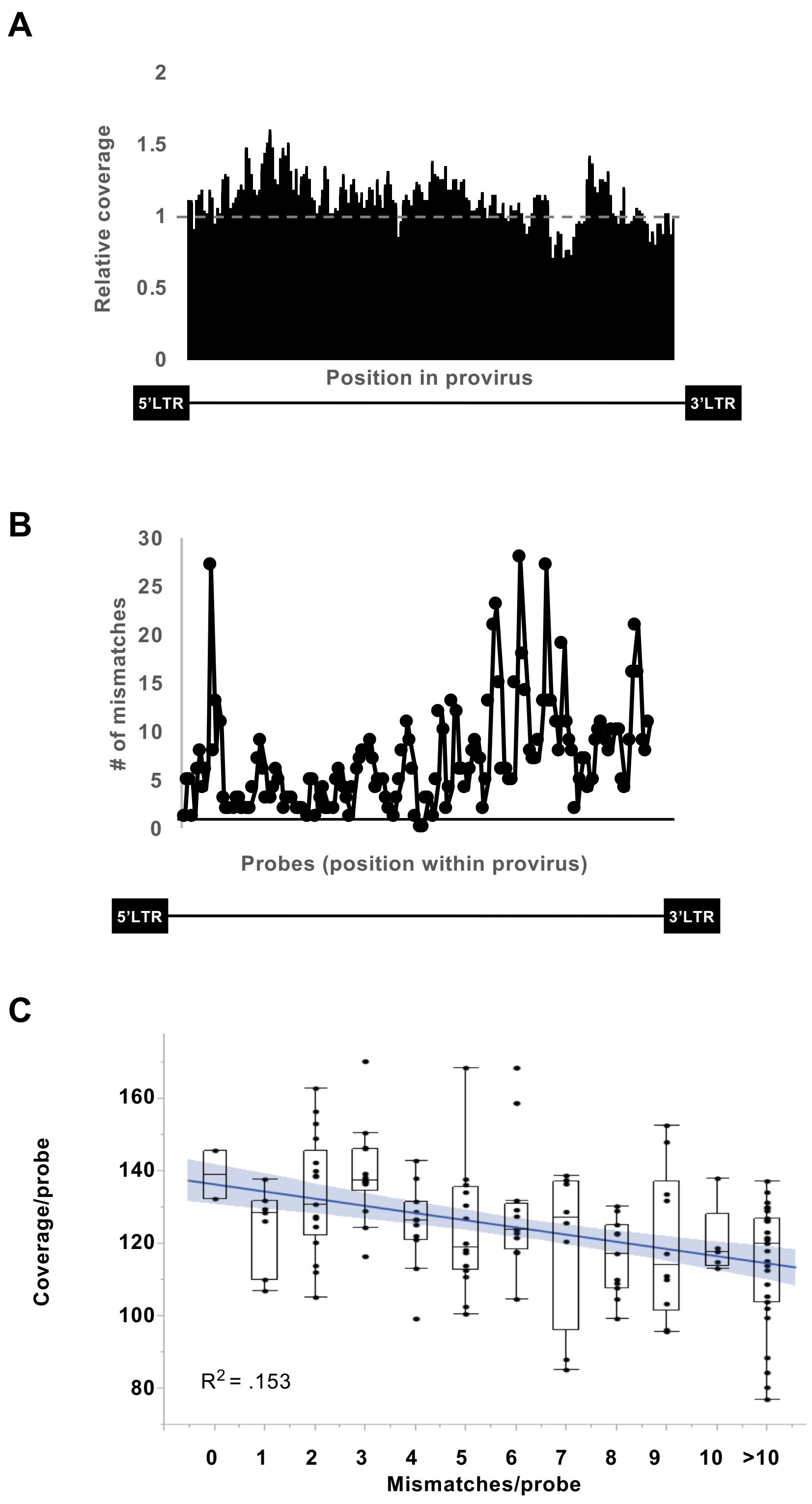

Supplementary Figure S2 HIV-1 DNA-capture-seq using U1 cells
A) Relative coverages of HIV-1 sequence in the HIV-1 DNA-capture-seq using U1 cells.
B) Number of nucleotide mismatches between HIV-1 provirus in U1 cells and DNA probes at each probe position.
C) A relationship between HIV-1 sequence coverage and number of sequence mismatch per each probe.

Table S1. A list of the chimeric reads detected in the analysis of mixed DNA of Jurkat cell and NL4-3 plasmid

IS_Id	ID	Chrom.left	Start.left	End.left	Reads.left	Chrom.right	Start.right	End.right	Reads.right	Reads.total	Strand
IS_0	1	chr1	3770245	3770321	1	.	.	.	.	1	+
IS_1	2	chr1	21384208	21384284	1	.	.	.	.	1	+
IS_2	3	chr1	22621184	22621260	1	.	.	.	.	1	-
IS_3	4	chr1	24948743	24948819	1	.	.	.	.	1	+
IS_4	5	chr1	24996589	24996665	1	.	.	.	.	1	-
IS_5	6	chr1	27897139	27897215	1	.	.	.	.	1	+
IS_6	7	chr1	39008437	39008513	1	.	.	.	.	1	+
IS_7	8	chr1	39556430	39556506	1	.	.	.	.	1	-
IS_8	9	chr1	46208409	46208485	1	.	.	.	.	1	-
IS_9	10	chr1	51222253	51222329	1	.	.	.	.	1	+
IS_10	11	chr1	60812052	60812128	1	.	.	.	.	1	+
IS_11	12	chr1	70640983	70641035	1	.	.	.	.	1	+
IS_12	13	chr1	71253744	71253820	1	.	.	.	.	1	+
IS_13	14	chr1	71605613	71605655	1	.	.	.	.	1	+
IS_14	15	chr1	73612325	73612401	1	.	.	.	.	1	-
IS_15	16	chr1	74475751	74475803	1	.	.	.	.	1	-
IS_16	17	chr1	74498812	74498888	1	.	.	.	.	1	+
IS_17	18	chr1	74633014	74633090	1	.	.	.	.	1	-
IS_18	19	chr1	77695724	77695800	1	.	.	.	.	1	+
IS_19	20	chr1	80213006	80213082	1	.	.	.	.	1	-
IS_20	21	chr1	83064367	83064416	1	.	.	.	.	1	+
IS_21	22	chr1	84499728	84499804	1	.	.	.	.	1	-
IS_22	23	chr1	87508004	87508080	1	.	.	.	.	1	-
IS_23	24	chr1	90547601	90547677	1	.	.	.	.	1	+
IS_24	25	chr1	93243343	93243419	1	.	.	.	.	1	+
IS_25	26	chr1	96483789	96483865	1	.	.	.	.	1	-
IS_26	27	chr1	102411482	102411558	1	.	.	.	.	1	-
IS_27	28	chr1	103217687	103217763	1	.	.	.	.	1	-
IS_28	29	chr1	116199225	116199301	1	.	.	.	.	1	+
IS_29	30	chr1	116804758	116804800	1	.	.	.	.	1	+
IS_30	31	chr1	118492165	118492209	1	.	.	.	.	1	+
IS_31	32	chr1	121485259	121485335	1	.	.	.	.	1	-
IS_32	33	chr1	151419836	151419885	1	.	.	.	.	1	+
IS_33	34	chr1	163587276	163587326	1	.	.	.	.	1	+
IS_34	35	chr1	169906073	169906145	1	.	.	.	.	1	+
IS_35	36	chr1	173536648	173536724	1	.	.	.	.	1	-
IS_36	37	chr1	176258787	176258863	1	.	.	.	.	1	-
IS_37	38	chr1	180188016	180188071	1	.	.	.	.	1	-
IS_38	39	chr1	192636221	192636297	1	.	.	.	.	1	-
IS_39	40	chr1	193807997	193808073	1	.	.	.	.	1	+
IS_40	41	chr1	195419404	195419480	1	.	.	.	.	1	+
IS_41	42	chr1	198417843	198417919	1	.	.	.	.	1	+
IS_42	43	chr1	202144764	202144840	1	.	.	.	.	1	+
IS_43	44	chr1	203780964	203781040	1	.	.	.	.	1	+
IS_44	45	chr1	204535643	204535719	1	.	.	.	.	1	-
IS_45	46	chr1	207430396	207430472	1	.	.	.	.	1	+
IS_46	47	chr1	211349490	211349566	1	.	.	.	.	1	+
IS_47	48	chr1	222199775	222199851	1	.	.	.	.	1	-
IS_48	49	chr1	228954513	228954589	1	.	.	.	.	1	-
IS_49	50	chr1	234504096	234504172	1	.	.	.	.	1	-
IS_50	51	chr1	241732446	241732522	1	.	.	.	.	1	+
IS_51	52	chr1	241816745	241816821	1	.	.	.	.	1	+
IS_52	53	chr1	242670324	242670401	1	.	.	.	.	1	-
IS_53	54	chr1	243135147	243135192	1	.	.	.	.	1	+
IS_54	55	chr1	248268452	248268511	1	.	.	.	.	1	+
IS_55	56	chr2	2759238	2759310	1	.	.	.	.	1	-

IS_56	57	chr2	4961815	4961891	1	.	.	.	.	1	+
IS_57	58	chr2	8136195	8136271	1	.	.	.	.	1	+
IS_58	59	chr2	8839855	8839931	1	.	.	.	.	1	+
IS_59	60	chr2	13609172	13609248	1	.	.	.	.	1	-
IS_60	61	chr2	18101182	18101258	1	.	.	.	.	1	+
IS_61	62	chr2	20549593	20549648	1	.	.	.	.	1	-
IS_62	63	chr2	22276881	22276957	1	.	.	.	.	1	+
IS_63	64	chr2	23437448	23437524	1	.	.	.	.	1	+
IS_64	65	chr2	28810983	28811059	1	.	.	.	.	1	-
IS_65	66	chr2	58162332	58162408	1	.	.	.	.	1	+
IS_66	67	chr2	61710802	61710878	1	.	.	.	.	1	-
IS_67	68	chr2	64275774	64275846	1	.	.	.	.	1	-
IS_68	69	chr2	67325821	67325897	1	.	.	.	.	1	+
IS_69	70	chr2	71712823	71712899	1	.	.	.	.	1	+
IS_70	71	chr2	72302821	72302897	1	.	.	.	.	1	+
IS_71	72	chr2	78579686	78579762	1	.	.	.	.	1	-
IS_72	73	chr2	80809070	80809146	1	.	.	.	.	1	-
IS_73	74	chr2	81402456	81402532	1	.	.	.	.	1	-
IS_74	75	chr2	89324597	89324673	1	.	.	.	.	1	+
IS_75	76	chr2	112862693	112862769	1	.	.	.	.	1	-
IS_76	77	chr2	125718815	125718891	1	.	.	.	.	1	-
IS_77	78	chr2	127142814	127142890	1	.	.	.	.	1	-
IS_78	79	chr2	140264444	140264520	1	.	.	.	.	1	+
IS_79	80	chr2	140823278	140823354	1	.	.	.	.	1	-
IS_80	81	chr2	142989974	142990050	1	.	.	.	.	1	+
IS_81	82	chr2	147709200	147709276	1	.	.	.	.	1	+
IS_82	83	chr2	148819362	148819438	1	.	.	.	.	1	+
IS_83	84	chr2	155208387	155208463	1	.	.	.	.	1	-
IS_84	85	chr2	155687514	155687590	1	.	.	.	.	1	+
IS_85	86	chr2	156005307	156005359	1	.	.	.	.	1	-
IS_86	87	chr2	157675466	157675542	1	.	.	.	.	1	+
IS_87	88	chr2	163528542	163528618	1	.	.	.	.	1	-
IS_88	89	chr2	177925055	177925115	1	.	.	.	.	1	+
IS_89	90	chr2	180879680	180879756	1	.	.	.	.	1	-
IS_90	91	chr2	183980480	183980556	1	.	.	.	.	1	+
IS_91	92	chr2	202262640	202262712	1	.	.	.	.	1	-
IS_92	93	chr2	205261418	205261494	1	.	.	.	.	1	-
IS_93	94	chr2	206165885	206165961	1	.	.	.	.	1	+
IS_94	95	chr2	208818298	208818374	1	.	.	.	.	1	+
IS_95	96	chr2	226828449	226828525	1	.	.	.	.	1	-
IS_96	97	chr2	230719099	230719153	1	.	.	.	.	1	-
IS_97	98	chr2	231695260	231695336	1	.	.	.	.	1	+
IS_98	99	chr2	233909759	233909835	1	.	.	.	.	1	+
IS_99	100	chr2	234441210	234441286	1	.	.	.	.	1	-
IS_100	101	chr2	234546311	234546387	1	.	.	.	.	1	+
IS_101	102	chr3	9270143	9270219	1	.	.	.	.	1	-
IS_102	103	chr3	9573682	9573758	1	.	.	.	.	1	-
IS_103	104	chr3	10148109	10148161	1	.	.	.	.	1	-
IS_104	105	chr3	12165717	12165793	1	.	.	.	.	1	+
IS_105	106	chr3	17075362	17075406	1	.	.	.	.	1	+
IS_106	107	chr3	17924426	17924493	1	.	.	.	.	1	+
IS_107	108	chr3	21509537	21509613	1	.	.	.	.	1	-
IS_108	109	chr3	26361534	26361610	1	.	.	.	.	1	-
IS_109	110	chr3	29536456	29536517	1	.	.	.	.	1	+
IS_110	111	chr3	34706414	34706473	1	.	.	.	.	1	-
IS_111	112	chr3	45655829	45655905	1	.	.	.	.	1	+
IS_112	113	chr3	50807578	50807641	1	.	.	.	.	1	+
IS_113	114	chr3	51390547	51390623	1	.	.	.	.	1	+
IS_114	115	chr3	54090769	54090845	1	.	.	.	.	1	+

IS_115	116	chr3	59652042	59652118	1	.	.	.	.	1	+
IS_116	117	chr3	68195197	68195273	1	.	.	.	.	1	+
IS_117	118	chr3	86472066	86472142	1	.	.	.	.	1	+
IS_118	119	chr3	88280965	88281041	1	.	.	.	.	1	-
IS_119	120	chr3	90454372	90454448	1	.	.	.	.	1	+
IS_120	121	chr3	96594634	96594710	1	.	.	.	.	1	-
IS_121	122	chr3	100923057	100923133	1	.	.	.	.	1	-
IS_122	123	chr3	101988752	101988828	1	.	.	.	.	1	+
IS_123	124	chr3	114051128	114051204	1	.	.	.	.	1	+
IS_124	125	chr3	124084773	124084849	1	.	.	.	.	1	-
IS_125	126	chr3	131557118	131557180	1	.	.	.	.	1	+
IS_126	127	chr3	133206516	133206592	1	.	.	.	.	1	+
IS_127	128	chr3	142444684	142444760	1	.	.	.	.	1	+
IS_128	129	chr3	153528753	153528829	1	.	.	.	.	1	+
IS_129	130	chr3	157466174	157466243	1	.	.	.	.	1	+
IS_130	131	chr3	161459742	161459798	1	.	.	.	.	1	+
IS_131	132	chr3	166168065	166168126	1	.	.	.	.	1	-
IS_132	133	chr3	172617522	172617584	1	.	.	.	.	1	-
IS_133	134	chr3	174307825	174307901	1	.	.	.	.	1	+
IS_134	135	chr3	178422842	178422918	1	.	.	.	.	1	-
IS_135	136	chr3	180916467	180916543	1	.	.	.	.	1	-
IS_136	137	chr3	183023286	183023362	1	.	.	.	.	1	-
IS_137	138	chr3	183171732	183171799	1	.	.	.	.	1	-
IS_138	139	chr3	188836968	188837044	1	.	.	.	.	1	+
IS_139	140	chr3	189767942	189768018	1	.	.	.	.	1	+
IS_140	141	chr3	196791168	196791244	1	.	.	.	.	1	-
IS_141	142	chr4	12847770	12847846	1	.	.	.	.	1	-
IS_142	143	chr4	22956111	22956177	1	.	.	.	.	1	+
IS_143	144	chr4	32782206	32782282	1	.	.	.	.	1	+
IS_144	145	chr4	33122598	33122674	1	.	.	.	.	1	+
IS_145	146	chr4	36022494	36022570	1	.	.	.	.	1	+
IS_146	147	chr4	39195145	39195221	1	.	.	.	.	1	-
IS_147	148	chr4	43813289	43813365	1	.	.	.	.	1	-
IS_148	149	chr4	44665377	44665453	1	.	.	.	.	1	-
IS_149	150	chr4	53704710	53704786	1	.	.	.	.	1	-
IS_150	151	chr4	60033100	60033176	1	.	.	.	.	1	+
IS_151	152	chr4	61315544	61315603	1	.	.	.	.	1	-
IS_152	153	chr4	68265891	68265939	1	.	.	.	.	1	+
IS_153	154	chr4	69037882	69037949	1	.	.	.	.	1	-
IS_154	155	chr4	71441606	71441682	1	.	.	.	.	1	-
IS_155	156	chr4	75259921	75259991	1	.	.	.	.	1	+
IS_156	157	chr4	75613881	75613957	1	.	.	.	.	1	-
IS_157	158	chr4	75728823	75728899	1	.	.	.	.	1	+
IS_158	159	chr4	78249935	78249993	1	.	.	.	.	1	-
IS_159	160	chr4	90145913	90145970	1	.	.	.	.	1	-
IS_160	161	chr4	109430412	109430488	1	.	.	.	.	1	-
IS_161	162	chr4	119932699	119932775	1	.	.	.	.	1	-
IS_162	163	chr4	120801410	120801486	1	.	.	.	.	1	-
IS_163	164	chr4	125233603	125233679	1	.	.	.	.	1	-
IS_164	165	chr4	139918715	139918791	1	.	.	.	.	1	-
IS_165	166	chr4	143309831	143309907	1	.	.	.	.	1	-
IS_166	167	chr4	144857557	144857633	1	.	.	.	.	1	+
IS_167	168	chr4	144924443	144924519	1	.	.	.	.	1	-
IS_168	169	chr4	153261617	153261693	1	.	.	.	.	1	-
IS_169	170	chr4	162489073	162489149	1	.	.	.	.	1	+
IS_170	171	chr4	163558319	163558395	1	.	.	.	.	1	+
IS_171	172	chr4	179149061	179149129	1	.	.	.	.	1	-
IS_172	173	chr4	182120977	182121053	1	.	.	.	.	1	-
IS_173	174	chr4	184330844	184330920	1	.	.	.	.	1	-

IS_174	175	chr4	186767272	186767348	1	.	.	.	.	1	-
IS_175	176	chr4	190665891	190665967	1	.	.	.	.	1	-
IS_176	177	chr5	11980300	11980349	1	.	.	.	.	1	+
IS_177	178	chr5	13227886	13227962	1	.	.	.	.	1	+
IS_178	179	chr5	17945953	17946029	1	.	.	.	.	1	-
IS_179	180	chr5	21636269	21636345	1	.	.	.	.	1	-
IS_180	181	chr5	24103702	24103767	1	.	.	.	.	1	+
IS_181	182	chr5	29898325	29898401	1	.	.	.	.	1	-
IS_182	183	chr5	30778345	30778421	1	.	.	.	.	1	-
IS_183	184	chr5	35634284	35634360	1	.	.	.	.	1	-
IS_184	185	chr5	37977023	37977099	1	.	.	.	.	1	-
IS_185	186	chr5	42260389	42260465	1	.	.	.	.	1	-
IS_186	187	chr5	43550297	43550373	1	.	.	.	.	1	-
IS_187	188	chr5	45185179	45185255	1	.	.	.	.	1	-
IS_188	189	chr5	45297898	45297974	1	.	.	.	.	1	-
IS_189	190	chr5	49640154	49640195	1	.	.	.	.	1	-
IS_190	191	chr5	61057307	61057383	1	.	.	.	.	1	-
IS_191	192	chr5	62309074	62309150	1	.	.	.	.	1	-
IS_192	193	chr5	65237133	65237220	1	.	.	.	.	1	+
IS_193	194	chr5	68086265	68086341	1	.	.	.	.	1	-
IS_194	195	chr5	105654104	105654180	1	.	.	.	.	1	+
IS_195	196	chr5	106525785	106525860	1	.	.	.	.	1	+
IS_196	197	chr5	109441526	109441602	1	.	.	.	.	1	+
IS_197	198	chr5	114691746	114691822	1	.	.	.	.	1	-
IS_198	199	chr5	118109160	118109236	1	.	.	.	.	1	+
IS_199	200	chr5	121366897	121366973	1	.	.	.	.	1	+
IS_200	201	chr5	131510875	131510951	1	.	.	.	.	1	+
IS_201	202	chr5	132067496	132067572	1	.	.	.	.	1	+
IS_202	203	chr5	134088773	134088847	1	.	.	.	.	1	-
IS_203	204	chr5	155956692	155956768	1	.	.	.	.	1	+
IS_204	205	chr5	156113803	156113879	1	.	.	.	.	1	-
IS_205	206	chr5	163698051	163698127	1	.	.	.	.	1	+
IS_206	207	chr5	167595162	167595224	1	.	.	.	.	1	-
IS_207	208	chr5	170618314	170618379	1	.	.	.	.	1	+
IS_208	209	chr5	173686060	173686136	1	.	.	.	.	1	-
IS_209	210	chr5	180360253	180360329	1	.	.	.	.	1	-
IS_210	211	chr6	8044629	8044705	1	.	.	.	.	1	+
IS_211	212	chr6	10471611	10471687	1	.	.	.	.	1	-
IS_212	213	chr6	22558728	22558804	1	.	.	.	.	1	+
IS_213	214	chr6	25767412	25767488	1	.	.	.	.	1	+
IS_214	215	chr6	35545977	35546053	1	.	.	.	.	1	-
IS_215	216	chr6	36504002	36504078	1	.	.	.	.	1	+
IS_216	217	chr6	38095978	38096054	1	.	.	.	.	1	-
IS_217	218	chr6	39837173	39837249	1	.	.	.	.	1	+
IS_218	219	chr6	41733989	41734065	1	.	.	.	.	1	+
IS_219	220	chr6	43330648	43330724	1	.	.	.	.	1	+
IS_220	221	chr6	47145666	47145725	1	.	.	.	.	1	-
IS_221	222	chr6	55810487	55810563	1	.	.	.	.	1	+
IS_222	223	chr6	64255245	64255321	1	.	.	.	.	1	-
IS_223	224	chr6	69008411	69008487	1	.	.	.	.	1	+
IS_224	225	chr6	87846100	87846176	1	.	.	.	.	1	+
IS_225	226	chr6	89390394	89390470	1	.	.	.	.	1	+
IS_226	227	chr6	90846581	90846657	1	.	.	.	.	1	+
IS_227	228	chr6	92126935	92127011	1	.	.	.	.	1	-
IS_228	229	chr6	95261880	95261956	1	.	.	.	.	1	-
IS_229	230	chr6	104283446	104283522	1	.	.	.	.	1	-
IS_230	231	chr6	112173428	112173504	1	.	.	.	.	1	-
IS_231	232	chr6	112333342	112333418	1	.	.	.	.	1	-
IS_232	233	chr6	115719283	115719359	1	.	.	.	.	1	-

IS_233	234	chr6	119766273	119766349	1	.	.	.	.	1	-
IS_234	235	chr6	120144867	120144943	1	.	.	.	.	1	+
IS_235	236	chr6	126121880	126121956	1	.	.	.	.	1	-
IS_236	237	chr6	131077295	131077371	1	.	.	.	.	1	-
IS_237	238	chr6	136983423	136983499	1	.	.	.	.	1	-
IS_238	239	chr6	137952546	137952622	1	.	.	.	.	1	-
IS_239	240	chr6	143418848	143418924	1	.	.	.	.	1	-
IS_240	241	chr6	145955738	145955814	1	.	.	.	.	1	+
IS_241	242	chr6	154383027	154383103	1	.	.	.	.	1	+
IS_242	243	chr6	155886871	155886947	1	.	.	.	.	1	+
IS_243	244	chr6	159352842	159352918	1	.	.	.	.	1	-
IS_244	245	chr6	163836603	163836679	1	.	.	.	.	1	-
IS_245	246	chr6	164989930	164990006	1	.	.	.	.	1	-
IS_246	247	chr6	165926370	165926417	1	.	.	.	.	1	+
IS_247	248	chr7	3958962	3959038	1	.	.	.	.	1	-
IS_248	249	chr7	7965175	7965251	1	.	.	.	.	1	+
IS_249	250	chr7	8207323	8207399	1	.	.	.	.	1	-
IS_250	251	chr7	12748382	12748458	1	.	.	.	.	1	-
IS_251	252	chr7	19225915	19225991	1	.	.	.	.	1	-
IS_252	253	chr7	22993417	22993493	1	.	.	.	.	1	+
IS_253	254	chr7	28713789	28713856	1	.	.	.	.	1	+
IS_254	255	chr7	30989135	30989211	1	.	.	.	.	1	-
IS_255	256	chr7	39276445	39276501	1	.	.	.	.	1	+
IS_256	257	chr7	44860481	44860557	1	.	.	.	.	1	-
IS_257	258	chr7	52029913	52029989	1	.	.	.	.	1	-
IS_258	259	chr7	54914785	54914839	1	.	.	.	.	1	-
IS_259	260	chr7	61969998	61970061	1	.	.	.	.	1	-
IS_260	261	chr7	66780748	66780824	1	.	.	.	.	1	-
IS_261	262	chr7	67569717	67569793	1	.	.	.	.	1	-
IS_262	263	chr7	73733570	73733646	1	.	.	.	.	1	-
IS_263	264	chr7	80585246	80585322	1	.	.	.	.	1	-
IS_264	265	chr7	83228637	83228689	1	.	.	.	.	1	+
IS_265	266	chr7	88199170	88199246	1	.	.	.	.	1	+
IS_266	267	chr7	90861947	90862023	1	.	.	.	.	1	-
IS_267	268	chr7	92488201	92488277	1	.	.	.	.	1	+
IS_268	269	chr7	94988391	94988467	1	.	.	.	.	1	-
IS_269	270	chr7	110829672	110829748	1	.	.	.	.	1	+
IS_270	271	chr7	111224019	111224095	1	.	.	.	.	1	-
IS_271	272	chr7	118855540	118855616	1	.	.	.	.	1	-
IS_272	273	chr7	122300100	122300176	1	.	.	.	.	1	+
IS_273	274	chr7	123960574	123960650	1	.	.	.	.	1	-
IS_274	275	chr7	127049068	127049144	1	.	.	.	.	1	-
IS_275	276	chr7	129448103	129448179	1	.	.	.	.	1	-
IS_276	277	chr7	132451223	132451299	1	.	.	.	.	1	-
IS_277	278	chr7	140356715	140356791	1	.	.	.	.	1	-
IS_278	279	chr7	151046444	151046520	1	.	.	.	.	1	+
IS_279	280	chr7	156862001	156862077	1	.	.	.	.	1	-
IS_280	281	chr7	157313527	157313603	1	.	.	.	.	1	+
IS_281	282	chr7	158488130	158488175	1	.	.	.	.	1	+
IS_282	283	chr8	1451660	1451736	1	.	.	.	.	1	+
IS_283	284	chr8	3903729	3903805	1	.	.	.	.	1	+
IS_284	285	chr8	14101389	14101465	1	.	.	.	.	1	+
IS_285	286	chr8	16169673	16169723	1	.	.	.	.	1	+
IS_286	287	chr8	16700933	16701009	1	.	.	.	.	1	+
IS_287	288	chr8	17337861	17337942	1	.	.	.	.	1	+
IS_288	289	chr8	21377357	21377433	1	.	.	.	.	1	+
IS_289	290	chr8	23948761	23948837	1	.	.	.	.	1	+
IS_290	291	chr8	26173310	26173386	1	.	.	.	.	1	-
IS_291	292	chr8	29225387	29225463	1	.	.	.	.	1	-

IS_292	293	chr8	30816426	30816502	1	.	.	.	.	1	-
IS_293	294	chr8	38998672	38998748	1	.	.	.	.	1	+
IS_294	295	chr8	39722332	39722408	1	.	.	.	.	1	+
IS_295	296	chr8	40988410	40988478	1	.	.	.	.	1	+
IS_296	297	chr8	47634181	47634249	1	.	.	.	.	1	+
IS_297	298	chr8	49131679	49131755	1	.	.	.	.	1	+
IS_298	299	chr8	51029483	51029559	1	.	.	.	.	1	+
IS_299	300	chr8	59290368	59290444	1	.	.	.	.	1	+
IS_300	301	chr8	61379452	61379528	1	.	.	.	.	1	+
IS_301	302	chr8	65374456	65374532	1	.	.	.	.	1	-
IS_302	303	chr8	71883689	71883765	1	.	.	.	.	1	-
IS_303	304	chr8	73931513	73931589	1	.	.	.	.	1	-
IS_304	305	chr8	80318153	80318229	1	.	.	.	.	1	+
IS_305	306	chr8	88719499	88719575	1	.	.	.	.	1	-
IS_306	307	chr8	91132439	91132508	1	.	.	.	.	1	+
IS_307	308	chr8	93474412	93474488	1	.	.	.	.	1	+
IS_308	309	chr8	94030994	94031070	1	.	.	.	.	1	+
IS_309	310	chr8	94391590	94391666	1	.	.	.	.	1	-
IS_310	311	chr8	99149443	99149519	1	.	.	.	.	1	-
IS_311	312	chr8	100583385	100583442	1	.	.	.	.	1	-
IS_312	313	chr8	100667056	100667133	1	.	.	.	.	1	+
IS_313	314	chr8	112720731	112720807	1	.	.	.	.	1	-
IS_314	315	chr8	115971984	115972060	1	.	.	.	.	1	+
IS_315	316	chr8	119236216	119236292	1	.	.	.	.	1	+
IS_316	317	chr8	124885614	124885690	1	.	.	.	.	1	+
IS_317	318	chr8	125008778	125008834	1	.	.	.	.	1	-
IS_318	319	chr8	126899842	126899918	1	.	.	.	.	1	-
IS_319	320	chr8	128633688	128633764	1	.	.	.	.	1	+
IS_320	321	chr8	134153392	134153468	1	.	.	.	.	1	+
IS_321	322	chr8	134964856	134964905	1	.	.	.	.	1	-
IS_322	323	chr8	137381146	137381222	1	.	.	.	.	1	-
IS_323	324	chr9	772095	772171	1	.	.	.	.	1	-
IS_324	325	chr9	15495068	15495144	1	.	.	.	.	1	-
IS_325	326	chr9	23343897	23343973	1	.	.	.	.	1	+
IS_326	327	chr9	31897654	31897730	1	.	.	.	.	1	-
IS_327	328	chr9	34679768	34679844	1	.	.	.	.	1	+
IS_328	329	chr9	37246896	37246972	1	.	.	.	.	1	-
IS_329	330	chr9	76505493	76505569	1	.	.	.	.	1	+
IS_330	331	chr9	76683357	76683433	1	.	.	.	.	1	-
IS_331	332	chr9	78432978	78433054	1	.	.	.	.	1	+
IS_332	333	chr9	85809117	85809193	1	.	.	.	.	1	-
IS_333	334	chr9	94286928	94286983	1	.	.	.	.	1	+
IS_334	335	chr9	95110270	95110346	1	.	.	.	.	1	+
IS_335	336	chr9	99360238	99360314	1	.	.	.	.	1	-
IS_336	337	chr9	100013873	100013949	1	.	.	.	.	1	+
IS_337	338	chr9	102024827	102024903	1	.	.	.	.	1	-
IS_338	339	chr9	109788856	109788932	1	.	.	.	.	1	-
IS_339	340	chr9	117713023	117713099	1	.	.	.	.	1	+
IS_340	341	chr9	120747482	120747531	1	.	.	.	.	1	-
IS_341	342	chr9	121284546	121284622	1	.	.	.	.	1	+
IS_342	343	chr9	124463712	124463788	1	.	.	.	.	1	+
IS_343	344	chr9	129813266	129813342	1	.	.	.	.	1	+
IS_344	345	chr9	134354159	134354235	1	.	.	.	.	1	+
IS_345	346	chr10	8238367	8238443	1	.	.	.	.	1	+
IS_346	347	chr10	11903571	11903647	1	.	.	.	.	1	-
IS_347	348	chr10	12749862	12749938	1	.	.	.	.	1	+
IS_348	349	chr10	13600536	13600594	1	.	.	.	.	1	+
IS_349	350	chr10	16538405	16538481	1	.	.	.	.	1	-
IS_350	351	chr10	17041182	17041258	1	.	.	.	.	1	+

IS_351	352	chr10	17746618	17746680	1	.	.	.	.	1	+
IS_352	353	chr10	31310474	31310537	1	.	.	.	.	1	-
IS_353	354	chr10	33452219	33452295	1	.	.	.	.	1	+
IS_354	355	chr10	33834303	33834379	1	.	.	.	.	1	-
IS_355	356	chr10	35449968	35450044	1	.	.	.	.	1	-
IS_356	357	chr10	36349587	36349660	1	.	.	.	.	1	+
IS_357	358	chr10	37842018	37842094	1	.	.	.	.	1	+
IS_358	359	chr10	43096867	43096928	1	.	.	.	.	1	-
IS_359	360	chr10	43942759	43942835	1	.	.	.	.	1	+
IS_360	361	chr10	44504477	44504553	1	.	.	.	.	1	-
IS_361	362	chr10	45039648	45039724	1	.	.	.	.	1	+
IS_362	363	chr10	48404628	48404704	1	.	.	.	.	1	+
IS_363	364	chr10	50443761	50443837	1	.	.	.	.	1	+
IS_364	365	chr10	52175178	52175246	1	.	.	.	.	1	+
IS_365	366	chr10	54431789	54431865	1	.	.	.	.	1	-
IS_366	367	chr10	56296269	56296345	1	.	.	.	.	1	-
IS_367	368	chr10	59931360	59931436	1	.	.	.	.	1	+
IS_368	369	chr10	60377932	60378008	1	.	.	.	.	1	+
IS_369	370	chr10	63179518	63179594	1	.	.	.	.	1	+
IS_370	371	chr10	63769064	63769140	1	.	.	.	.	1	+
IS_371	372	chr10	64213761	64213808	1	.	.	.	.	1	-
IS_372	373	chr10	64980520	64980596	1	.	.	.	.	1	+
IS_373	374	chr10	67952322	67952398	1	.	.	.	.	1	+
IS_374	375	chr10	74248019	74248095	1	.	.	.	.	1	+
IS_375	376	chr10	74831416	74831492	1	.	.	.	.	1	+
IS_376	377	chr10	76275795	76275871	1	.	.	.	.	1	+
IS_377	378	chr10	81879982	81880058	1	.	.	.	.	1	-
IS_378	379	chr10	82761226	82761302	1	.	.	.	.	1	+
IS_379	380	chr10	84697160	84697236	1	.	.	.	.	1	-
IS_380	381	chr10	84721826	84721902	1	.	.	.	.	1	+
IS_381	382	chr10	98915872	98915948	1	.	.	.	.	1	+
IS_382	383	chr10	105222074	105222123	1	.	.	.	.	1	-
IS_383	384	chr10	106432267	106432308	1	.	.	.	.	1	+
IS_384	385	chr10	115089315	115089391	1	.	.	.	.	1	-
IS_385	386	chr10	118801312	118801388	1	.	.	.	.	1	+
IS_386	387	chr10	119538648	119538724	1	.	.	.	.	1	+
IS_387	388	chr10	120614402	120614478	1	.	.	.	.	1	+
IS_388	389	chr11	6798834	6798910	1	.	.	.	.	1	+
IS_389	390	chr11	10241555	10241631	1	.	.	.	.	1	-
IS_390	391	chr11	15148481	15148557	1	.	.	.	.	1	+
IS_391	392	chr11	22904293	22904359	1	.	.	.	.	1	-
IS_392	393	chr11	23218463	23218539	1	.	.	.	.	1	-
IS_393	394	chr11	27467316	27467387	1	.	.	.	.	1	-
IS_394	395	chr11	40266679	40266748	1	.	.	.	.	1	+
IS_395	396	chr11	42858430	42858506	1	.	.	.	.	1	+
IS_396	397	chr11	44929919	44929995	1	.	.	.	.	1	-
IS_397	398	chr11	46118999	46119075	1	.	.	.	.	1	-
IS_398	399	chr11	58435233	58435309	1	.	.	.	.	1	-
IS_399	400	chr11	78973796	78973872	1	.	.	.	.	1	+
IS_400	401	chr11	82433425	82433501	1	.	.	.	.	1	+
IS_401	402	chr11	82896030	82896106	1	.	.	.	.	1	-
IS_402	403	chr11	83330640	83330716	1	.	.	.	.	1	+
IS_403	404	chr11	83711781	83711857	1	.	.	.	.	1	-
IS_404	405	chr11	84890132	84890208	1	.	.	.	.	1	+
IS_405	406	chr11	85729832	85729908	1	.	.	.	.	1	-
IS_406	407	chr11	86829685	86829744	1	.	.	.	.	1	-
IS_407	408	chr11	87058064	87058118	1	.	.	.	.	1	-
IS_408	409	chr11	90899495	90899571	1	.	.	.	.	1	+
IS_409	410	chr11	97499622	97499698	1	.	.	.	.	1	-

IS_410	411	chr11	106742857	106742933	1	.	.	.	.	1	+
IS_411	412	chr11	109080391	109080467	1	.	.	.	.	1	-
IS_412	413	chr11	109661817	109661893	1	.	.	.	.	1	-
IS_413	414	chr11	119709604	119709680	1	.	.	.	.	1	-
IS_414	415	chr11	121724086	121724162	1	.	.	.	.	1	-
IS_415	416	chr11	126601022	126601098	1	.	.	.	.	1	-
IS_416	417	chr11	130213911	130213967	1	.	.	.	.	1	+
IS_417	418	chr12	9940379	9940455	1	.	.	.	.	1	+
IS_418	419	chr12	10476108	10476184	1	.	.	.	.	1	+
IS_419	420	chr12	13630438	13630514	1	.	.	.	.	1	+
IS_420	421	chr12	23203343	23203419	1	.	.	.	.	1	-
IS_421	422	chr12	24624766	24624843	1	.	.	.	.	1	-
IS_422	423	chr12	34510321	34510397	1	.	.	.	.	1	-
IS_423	424	chr12	46878460	46878536	1	.	.	.	.	1	-
IS_424	425	chr12	82122644	82122720	1	.	.	.	.	1	-
IS_425	426	chr12	85307750	85307826	1	.	.	.	.	1	+
IS_426	427	chr12	96540271	96540347	1	.	.	.	.	1	+
IS_427	428	chr12	104778402	104778478	1	.	.	.	.	1	-
IS_428	429	chr12	107586184	107586260	1	.	.	.	.	1	+
IS_429	430	chr12	113157847	113157923	1	.	.	.	.	1	-
IS_430	431	chr12	113722021	113722097	1	.	.	.	.	1	+
IS_431	432	chr12	127301946	127302022	1	.	.	.	.	1	-
IS_432	433	chr12	127631252	127631328	1	.	.	.	.	1	-
IS_433	434	chr12	129422857	129422933	1	.	.	.	.	1	-
IS_434	435	chr12	131449208	131449244	1	.	.	.	.	1	+
IS_435	436	chr13	20471282	20471351	1	.	.	.	.	1	-
IS_436	437	chr13	20491920	20491974	1	.	.	.	.	1	+
IS_437	438	chr13	26354760	26354815	1	.	.	.	.	1	+
IS_438	439	chr13	27381592	27381668	1	.	.	.	.	1	-
IS_439	440	chr13	36716563	36716639	1	.	.	.	.	1	+
IS_440	441	chr13	38552856	38552932	1	.	.	.	.	1	+
IS_441	442	chr13	63519576	63519652	1	.	.	.	.	1	+
IS_442	443	chr13	68491985	68492061	1	.	.	.	.	1	+
IS_443	444	chr13	72930892	72930968	1	.	.	.	.	1	+
IS_444	445	chr13	74079860	74079922	1	.	.	.	.	1	-
IS_445	446	chr13	75090914	75090959	1	.	.	.	.	1	-
IS_446	447	chr13	78733262	78733338	1	.	.	.	.	1	-
IS_447	448	chr13	83719344	83719420	1	.	.	.	.	1	+
IS_448	449	chr13	89382176	89382248	1	.	.	.	.	1	+
IS_449	450	chr13	93532912	93532988	1	.	.	.	.	1	+
IS_450	451	chr13	100510429	100510505	1	.	.	.	.	1	-
IS_451	452	chr13	103276725	103276801	1	.	.	.	.	1	-
IS_452	453	chr13	108207214	108207279	1	.	.	.	.	1	+
IS_453	454	chr13	111409652	111409728	1	.	.	.	.	1	+
IS_454	455	chr13	111610095	111610171	1	.	.	.	.	1	-
IS_455	456	chr14	35439073	35439149	1	.	.	.	.	1	+
IS_456	457	chr14	35762638	35762714	1	.	.	.	.	1	-
IS_457	458	chr14	41703325	41703401	1	.	.	.	.	1	-
IS_458	459	chr14	41931370	41931446	1	.	.	.	.	1	-
IS_459	460	chr14	44814815	44814891	1	.	.	.	.	1	-
IS_460	461	chr14	45686257	45686333	1	.	.	.	.	1	+
IS_461	462	chr14	49424709	49424785	1	.	.	.	.	1	+
IS_462	463	chr14	56430708	56430784	1	.	.	.	.	1	-
IS_463	464	chr14	58063353	58063429	2	.	.	.	.	2	+
IS_464	465	chr14	63506234	63506289	1	.	.	.	.	1	+
IS_465	466	chr14	71028392	71028468	1	.	.	.	.	1	-
IS_466	467	chr14	73558426	73558502	1	.	.	.	.	1	-
IS_467	468	chr14	76244355	76244431	1	.	.	.	.	1	-
IS_468	469	chr14	85565470	85565546	1	.	.	.	.	1	+

IS_469	470	chr14	94132499	94132567	1	.	.	.	.	1	+
IS_470	471	chr14	96056794	96056870	1	.	.	.	.	1	+
IS_471	472	chr14	102138827	102138903	1	.	.	.	.	1	-
IS_472	473	chr15	27818422	27818498	1	.	.	.	.	1	+
IS_473	474	chr15	31603178	31603254	1	.	.	.	.	1	-
IS_474	475	chr15	37459206	37459261	1	.	.	.	.	1	+
IS_475	476	chr15	43131055	43131131	1	.	.	.	.	1	-
IS_476	477	chr15	44806166	44806242	1	.	.	.	.	1	+
IS_477	478	chr15	44933778	44933852	1	.	.	.	.	1	+
IS_478	479	chr15	45328324	45328400	1	.	.	.	.	1	-
IS_479	480	chr15	49750887	49750963	1	.	.	.	.	1	-
IS_480	481	chr15	50670738	50670814	1	.	.	.	.	1	+
IS_481	482	chr15	66440315	66440391	1	.	.	.	.	1	-
IS_482	483	chr15	70482470	70482546	1	.	.	.	.	1	-
IS_483	484	chr15	72058992	72059068	1	.	.	.	.	1	+
IS_484	485	chr15	73168211	73168287	1	.	.	.	.	1	-
IS_485	486	chr15	75392707	75392783	1	.	.	.	.	1	+
IS_486	487	chr15	80833089	80833165	1	.	.	.	.	1	+
IS_487	488	chr15	92321927	92322003	1	.	.	.	.	1	-
IS_488	489	chr15	92402792	92402868	1	.	.	.	.	1	+
IS_489	490	chr15	100742754	100742827	1	.	.	.	.	1	-
IS_490	491	chr16	366450	367156	3880	.	.	.	.	3880	+
IS_491	492	chr16	4591158	4591234	1	.	.	.	.	1	+
IS_492	493	chr16	4764500	4764576	1	.	.	.	.	1	+
IS_493	494	chr16	6394871	6394947	1	.	.	.	.	1	-
IS_494	495	chr16	9282287	9282331	1	.	.	.	.	1	-
IS_495	496	chr16	11288492	11288568	1	.	.	.	.	1	-
IS_496	497	chr16	20406060	20406136	1	.	.	.	.	1	-
IS_497	498	chr16	28316464	28316522	1	.	.	.	.	1	+
IS_498	499	chr16	53189117	53189188	1	.	.	.	.	1	+
IS_499	500	chr16	53703852	53703928	1	.	.	.	.	1	-
IS_500	501	chr16	55319442	55319518	1	.	.	.	.	1	-
IS_501	502	chr16	56516568	56516644	1	.	.	.	.	1	+
IS_502	503	chr16	59362055	59362131	1	.	.	.	.	1	-
IS_503	504	chr16	66507299	66507375	1	.	.	.	.	1	+
IS_504	505	chr16	84449739	84449803	1	.	.	.	.	1	+
IS_505	506	chr17	1256427	1256503	1	.	.	.	.	1	+
IS_506	507	chr17	1289731	1289807	1	.	.	.	.	1	-
IS_507	508	chr17	8466736	8466812	1	.	.	.	.	1	+
IS_508	509	chr17	12176980	12177018	1	.	.	.	.	1	+
IS_509	510	chr17	20739506	20739582	1	.	.	.	.	1	-
IS_510	511	chr17	22189146	22189222	1	.	.	.	.	1	-
IS_511	512	chr17	29108765	29108841	1	.	.	.	.	1	-
IS_512	513	chr17	31629717	31629793	1	.	.	.	.	1	+
IS_513	514	chr17	35163141	35163208	1	.	.	.	.	1	-
IS_514	515	chr17	42777447	42777523	1	.	.	.	.	1	+
IS_515	516	chr17	44356625	44356701	1	.	.	.	.	1	+
IS_516	517	chr17	46063466	46063542	1	.	.	.	.	1	+
IS_517	518	chr17	53733959	53734035	1	.	.	.	.	1	-
IS_518	519	chr17	57137494	57137570	1	.	.	.	.	1	+
IS_519	520	chr17	57233347	57233411	1	.	.	.	.	1	-
IS_520	521	chr17	57375361	57375437	1	.	.	.	.	1	-
IS_521	522	chr17	76628217	76628293	1	.	.	.	.	1	+
IS_522	523	chr17	80165536	80166231	8135	.	.	.	.	8135	-
IS_523	524	chr18	137705	137781	1	.	.	.	.	1	+
IS_524	525	chr18	6708490	6708566	1	.	.	.	.	1	+
IS_525	526	chr18	7245159	7245235	1	.	.	.	.	1	+
IS_526	527	chr18	12248011	12248087	1	.	.	.	.	1	+
IS_527	528	chr18	12634273	12634349	1	.	.	.	.	1	-

IS_528	529	chr18	18519279	18519355	1	.	.	.	.	1	-
IS_529	530	chr18	25538651	25538727	1	.	.	.	.	1	-
IS_530	531	chr18	28618373	28618449	1	.	.	.	.	1	+
IS_531	532	chr18	30449343	30449419	1	.	.	.	.	1	-
IS_532	533	chr18	34358928	34359004	1	.	.	.	.	1	-
IS_533	534	chr18	34475426	34475502	1	.	.	.	.	1	-
IS_534	535	chr18	36586244	36586320	1	.	.	.	.	1	-
IS_535	536	chr18	42178294	42178333	1	.	.	.	.	1	+
IS_536	537	chr18	53678729	53678805	1	.	.	.	.	1	+
IS_537	538	chr18	59678521	59678597	1	.	.	.	.	1	-
IS_538	539	chr18	69394858	69394934	1	.	.	.	.	1	-
IS_539	540	chr18	75613318	75613394	1	.	.	.	.	1	-
IS_540	541	chr19	2039458	2039534	1	.	.	.	.	1	-
IS_541	542	chr19	2490274	2490350	1	.	.	.	.	1	-
IS_542	543	chr19	20173176	20173226	1	.	.	.	.	1	+
IS_543	544	chr19	31593974	31594050	1	.	.	.	.	1	-
IS_544	545	chr19	38696497	38696573	1	.	.	.	.	1	+
IS_545	546	chr19	45481100	45481176	1	.	.	.	.	1	-
IS_546	547	chr19	50243504	50243580	1	.	.	.	.	1	+
IS_547	548	chr19	56831442	56831518	1	.	.	.	.	1	+
IS_548	549	chr19	58215625	58215694	1	.	.	.	.	1	+
IS_549	550	chr20	4783926	4784002	1	.	.	.	.	1	-
IS_550	551	chr20	5753890	5753966	1	.	.	.	.	1	+
IS_551	552	chr20	5835158	5835234	1	.	.	.	.	1	+
IS_552	553	chr20	21012077	21012153	1	.	.	.	.	1	+
IS_553	554	chr20	33370306	33370382	1	.	.	.	.	1	-
IS_554	555	chr20	41842238	41842289	1	.	.	.	.	1	+
IS_555	556	chr20	44178425	44178501	1	.	.	.	.	1	-
IS_556	557	chr20	45104128	45104204	1	.	.	.	.	1	-
IS_557	558	chr20	55879900	55879976	1	.	.	.	.	1	-
IS_558	559	chr21	10702699	10702771	1	.	.	.	.	1	-
IS_559	560	chr21	18696610	18696686	1	.	.	.	.	1	+
IS_560	561	chr21	25201314	25201390	1	.	.	.	.	1	+
IS_561	562	chr21	44950235	44950311	1	.	.	.	.	1	+
IS_562	563	chr21	45577689	45577765	1	.	.	.	.	1	-
IS_563	564	chr22	25661609	25661685	1	.	.	.	.	1	+
IS_564	565	chr22	29483552	29483630	1	.	.	.	.	1	-
IS_565	566	chr22	44605443	44605519	1	.	.	.	.	1	-
IS_566	567	chrX	5575430	5575506	1	.	.	.	.	1	+
IS_567	568	chrX	7550849	7550925	1	.	.	.	.	1	+
IS_568	569	chrX	11426590	11426666	1	.	.	.	.	1	-
IS_569	570	chrX	17129720	17129796	1	.	.	.	.	1	-
IS_570	571	chrX	21333543	21333619	1	.	.	.	.	1	+
IS_571	572	chrX	31325517	31325577	1	.	.	.	.	1	+
IS_572	573	chrX	38694620	38694696	1	.	.	.	.	1	+
IS_573	574	chrX	40108310	40108386	1	.	.	.	.	1	+
IS_574	575	chrX	41850256	41850332	1	.	.	.	.	1	-
IS_575	576	chrX	42347696	42347772	1	.	.	.	.	1	-
IS_576	577	chrX	49417449	49417525	1	.	.	.	.	1	-
IS_577	578	chrX	50530758	50530836	1	.	.	.	.	1	-
IS_578	579	chrX	69061135	69061211	1	.	.	.	.	1	+
IS_579	580	chrX	70677110	70677186	1	.	.	.	.	1	-
IS_580	581	chrX	109159508	109159584	1	.	.	.	.	1	-
IS_581	582	chrX	123679519	123679595	1	.	.	.	.	1	-
IS_582	583	chrX	126603543	126603583	1	.	.	.	.	1	+
IS_583	584	chrX	127009519	127009575	1	.	.	.	.	1	+
IS_584	585	.	.	.	.	chr1	2511387	2511463	1	1	-
IS_585	586	.	.	.	.	chr1	17090192	17090268	1	1	-
IS_586	587	.	.	.	.	chr1	21254030	21254106	1	1	-

IS_587	588	.	.	.	.	chr1	24711071	24711147	1	1	-
IS_588	589	.	.	.	.	chr1	29596786	29596862	1	1	+
IS_589	590	.	.	.	.	chr1	30581305	30581377	1	1	+
IS_590	591	.	.	.	.	chr1	30858451	30858527	1	1	+
IS_591	592	.	.	.	.	chr1	31751804	31751880	1	1	-
IS_592	593	.	.	.	.	chr1	36553213	36553289	1	1	+
IS_593	594	.	.	.	.	chr1	39694943	39694991	1	1	+
IS_594	595	.	.	.	.	chr1	51089605	51089681	1	1	-
IS_595	596	.	.	.	.	chr1	60111324	60111394	1	1	+
IS_596	597	.	.	.	.	chr1	61121034	61121110	1	1	+
IS_597	598	.	.	.	.	chr1	74161928	74161988	1	1	-
IS_598	599	.	.	.	.	chr1	77376179	77376255	1	1	+
IS_599	600	.	.	.	.	chr1	80580088	80580160	1	1	+
IS_600	601	.	.	.	.	chr1	81161243	81161319	1	1	-
IS_601	602	.	.	.	.	chr1	81861729	81861805	1	1	+
IS_602	603	.	.	.	.	chr1	85918213	85918268	1	1	+
IS_603	604	.	.	.	.	chr1	90586351	90586398	1	1	-
IS_604	605	.	.	.	.	chr1	91376977	91377053	1	1	-
IS_605	606	.	.	.	.	chr1	103162359	103162435	1	1	+
IS_606	607	.	.	.	.	chr1	103655608	103655684	1	1	-
IS_607	608	.	.	.	.	chr1	110948518	110948594	1	1	+
IS_608	609	.	.	.	.	chr1	111679855	111679935	1	1	+
IS_609	610	.	.	.	.	chr1	121484918	121484994	1	1	+
IS_610	611	.	.	.	.	chr1	147708339	147708415	1	1	+
IS_611	612	.	.	.	.	chr1	154444225	154444289	1	1	+
IS_612	613	.	.	.	.	chr1	154667579	154667635	1	1	+
IS_613	614	.	.	.	.	chr1	154680325	154680401	1	1	-
IS_614	615	.	.	.	.	chr1	155847019	155847095	1	1	-
IS_615	616	.	.	.	.	chr1	159276390	159276466	1	1	-
IS_616	617	.	.	.	.	chr1	161262102	161262178	1	1	+
IS_617	618	.	.	.	.	chr1	164930160	164930213	1	1	-
IS_618	619	.	.	.	.	chr1	171336488	171336564	1	1	-
IS_619	620	.	.	.	.	chr1	175644327	175644403	1	1	+
IS_620	621	.	.	.	.	chr1	176959632	176959677	1	1	-
IS_621	622	.	.	.	.	chr1	187620837	187620913	1	1	+
IS_622	623	.	.	.	.	chr1	191715008	191715084	1	1	+
IS_623	624	.	.	.	.	chr1	200594576	200594652	1	1	+
IS_624	625	.	.	.	.	chr1	201715561	201715614	1	1	-
IS_625	626	.	.	.	.	chr1	205745081	205745157	1	1	-
IS_626	627	.	.	.	.	chr1	213388506	213388582	1	1	+
IS_627	628	.	.	.	.	chr1	215358840	215358916	1	1	+
IS_628	629	.	.	.	.	chr1	230904498	230904574	1	1	-
IS_629	630	.	.	.	.	chr1	233489747	233489801	1	1	-
IS_630	631	.	.	.	.	chr1	234979873	234979949	1	1	-
IS_631	632	.	.	.	.	chr1	235691429	235691501	1	1	+
IS_632	633	.	.	.	.	chr1	238427745	238427821	1	1	-
IS_633	634	.	.	.	.	chr1	238906218	238906294	1	1	+
IS_634	635	.	.	.	.	chr1	245178042	245178118	1	1	+
IS_635	636	.	.	.	.	chr1	248199093	248199169	1	1	+
IS_636	637	.	.	.	.	chr2	2298478	2298554	1	1	-
IS_637	638	.	.	.	.	chr2	9589842	9589918	1	1	+
IS_638	639	.	.	.	.	chr2	16894791	16894867	1	1	+
IS_639	640	.	.	.	.	chr2	20141201	20141277	1	1	-
IS_640	641	.	.	.	.	chr2	25272953	25273031	1	1	-
IS_641	642	.	.	.	.	chr2	32475652	32475728	1	1	-
IS_642	643	.	.	.	.	chr2	33011760	33011827	1	1	+
IS_643	644	.	.	.	.	chr2	33141319	33141395	1	1	-
IS_644	645	.	.	.	.	chr2	39785956	39786032	1	1	+
IS_645	646	.	.	.	.	chr2	51956785	51956861	1	1	-

IS_646	647	.	.	.	.	chr2	54083723	54083777	1	1	-
IS_647	648	.	.	.	.	chr2	54642431	54642507	1	1	+
IS_648	649	.	.	.	.	chr2	55974098	55974161	1	1	-
IS_649	650	.	.	.	.	chr2	61727989	61728065	1	1	-
IS_650	651	.	.	.	.	chr2	66225640	66225716	1	1	+
IS_651	652	.	.	.	.	chr2	96328913	96328989	1	1	-
IS_652	653	.	.	.	.	chr2	101379403	101379454	1	1	+
IS_653	654	.	.	.	.	chr2	108013411	108013487	1	1	-
IS_654	655	.	.	.	.	chr2	114056730	114056806	1	1	-
IS_655	656	.	.	.	.	chr2	117034448	117034524	1	1	+
IS_656	657	.	.	.	.	chr2	120609668	120609744	1	1	+
IS_657	658	.	.	.	.	chr2	130375906	130375982	1	1	+
IS_658	659	.	.	.	.	chr2	137515671	137515747	1	1	+
IS_659	660	.	.	.	.	chr2	138838132	138838208	1	1	-
IS_660	661	.	.	.	.	chr2	150099106	150099182	1	1	-
IS_661	662	.	.	.	.	chr2	154419687	154419763	1	1	-
IS_662	663	.	.	.	.	chr2	155204689	155204765	1	1	-
IS_663	664	.	.	.	.	chr2	156598126	156598202	1	1	-
IS_664	665	.	.	.	.	chr2	160569619	160569689	1	1	+
IS_665	666	.	.	.	.	chr2	162351033	162351106	1	1	-
IS_666	667	.	.	.	.	chr2	176941677	176941753	1	1	+
IS_667	668	.	.	.	.	chr2	181237058	181237134	1	1	+
IS_668	669	.	.	.	.	chr2	182450813	182450889	1	1	+
IS_669	670	.	.	.	.	chr2	189698414	189698490	1	1	-
IS_670	671	.	.	.	.	chr2	189797060	189797119	1	1	-
IS_671	672	.	.	.	.	chr2	198705479	198705555	1	1	-
IS_672	673	.	.	.	.	chr2	200413367	200413444	1	1	-
IS_673	674	.	.	.	.	chr2	203233125	203233199	1	1	-
IS_674	675	.	.	.	.	chr2	206039316	206039392	1	1	-
IS_675	676	.	.	.	.	chr2	207119691	207119767	1	1	-
IS_676	677	.	.	.	.	chr2	207629448	207629524	1	1	-
IS_677	678	.	.	.	.	chr2	211083693	211083769	1	1	-
IS_678	679	.	.	.	.	chr2	217263959	217264035	1	1	+
IS_679	680	.	.	.	.	chr2	218026180	218026256	1	1	+
IS_680	681	.	.	.	.	chr2	222058764	222058841	1	1	+
IS_681	682	.	.	.	.	chr2	222103846	222103922	1	1	+
IS_682	683	.	.	.	.	chr2	222183047	222183123	1	1	-
IS_683	684	.	.	.	.	chr2	227061981	227062057	1	1	+
IS_684	685	.	.	.	.	chr2	227278282	227278358	1	1	+
IS_685	686	.	.	.	.	chr2	230440532	230440608	1	1	-
IS_686	687	.	.	.	.	chr2	241547284	241547335	1	1	-
IS_687	688	.	.	.	.	chr2	242896353	242896429	1	1	-
IS_688	689	.	.	.	.	chr3	7036020	7036096	1	1	-
IS_689	690	.	.	.	.	chr3	7921180	7921251	1	1	-
IS_690	691	.	.	.	.	chr3	10604864	10604905	1	1	-
IS_691	692	.	.	.	.	chr3	13785741	13785817	1	1	+
IS_692	693	.	.	.	.	chr3	16109287	16109363	1	1	+
IS_693	694	.	.	.	.	chr3	22529434	22529510	1	1	-
IS_694	695	.	.	.	.	chr3	32121792	32121868	1	1	+
IS_695	696	.	.	.	.	chr3	36837044	36837110	1	1	+
IS_696	697	.	.	.	.	chr3	36959110	36959186	1	1	-
IS_697	698	.	.	.	.	chr3	39585268	39585330	1	1	-
IS_698	699	.	.	.	.	chr3	40618007	40618083	1	1	-
IS_699	700	.	.	.	.	chr3	47767779	47767855	1	1	+
IS_700	701	.	.	.	.	chr3	52905765	52905841	1	1	+
IS_701	702	.	.	.	.	chr3	59234978	59235054	1	1	-
IS_702	703	.	.	.	.	chr3	62554618	62554694	1	1	+
IS_703	704	.	.	.	.	chr3	63060754	63060830	1	1	+
IS_704	705	.	.	.	.	chr3	66352046	66352122	1	1	+

IS_705	706	.	.	.	.	chr3	83891600	83891676	1	1	-
IS_706	707	.	.	.	.	chr3	97144662	97144738	1	1	-
IS_707	708	.	.	.	.	chr3	98397162	98397221	1	1	-
IS_708	709	.	.	.	.	chr3	100037063	100037134	1	1	+
IS_709	710	.	.	.	.	chr3	108074689	108074752	1	1	-
IS_710	711	.	.	.	.	chr3	121777897	121777973	1	1	+
IS_711	712	.	.	.	.	chr3	129575392	129575468	1	1	+
IS_712	713	.	.	.	.	chr3	132018020	132018057	1	1	-
IS_713	714	.	.	.	.	chr3	137979345	137979421	1	1	-
IS_714	715	.	.	.	.	chr3	148934086	148934162	1	1	+
IS_715	716	.	.	.	.	chr3	162320872	162320948	1	1	+
IS_716	717	.	.	.	.	chr3	170489791	170489867	1	1	+
IS_717	718	.	.	.	.	chr3	173637294	173637370	1	1	+
IS_718	719	.	.	.	.	chr3	174568236	174568312	1	1	-
IS_719	720	.	.	.	.	chr3	176453936	176454012	1	1	+
IS_720	721	.	.	.	.	chr3	176897543	176897619	1	1	-
IS_721	722	.	.	.	.	chr3	188119614	188119690	1	1	+
IS_722	723	.	.	.	.	chr3	190437039	190437115	1	1	-
IS_723	724	.	.	.	.	chr3	193148232	193148290	1	1	+
IS_724	725	.	.	.	.	chr3	196699423	196699499	1	1	+
IS_725	726	.	.	.	.	chr4	3123971	3124047	1	1	+
IS_726	727	.	.	.	.	chr4	15487190	15487266	1	1	-
IS_727	728	.	.	.	.	chr4	17427296	17427372	1	1	-
IS_728	729	.	.	.	.	chr4	20401290	20401366	1	1	-
IS_729	730	.	.	.	.	chr4	25863201	25863279	1	1	-
IS_730	731	.	.	.	.	chr4	28487708	28487784	1	1	+
IS_731	732	.	.	.	.	chr4	37744850	37744926	1	1	-
IS_732	733	.	.	.	.	chr4	46737939	46738015	1	1	+
IS_733	734	.	.	.	.	chr4	55906897	55906973	1	1	-
IS_734	735	.	.	.	.	chr4	56848927	56849003	1	1	-
IS_735	736	.	.	.	.	chr4	57514643	57514719	1	1	+
IS_736	737	.	.	.	.	chr4	58855000	58855076	1	1	+
IS_737	738	.	.	.	.	chr4	66447062	66447138	1	1	-
IS_738	739	.	.	.	.	chr4	68249033	68249109	1	1	-
IS_739	740	.	.	.	.	chr4	74473361	74473437	1	1	+
IS_740	741	.	.	.	.	chr4	81677171	81677235	1	1	-
IS_741	742	.	.	.	.	chr4	84007047	84007123	1	1	-
IS_742	743	.	.	.	.	chr4	98191485	98191561	1	1	+
IS_743	744	.	.	.	.	chr4	101834068	101834144	1	1	-
IS_744	745	.	.	.	.	chr4	107096954	107097030	1	1	-
IS_745	746	.	.	.	.	chr4	109559252	109559328	1	1	+
IS_746	747	.	.	.	.	chr4	110189547	110189623	1	1	-
IS_747	748	.	.	.	.	chr4	114366966	114367042	1	1	+
IS_748	749	.	.	.	.	chr4	118279837	118279897	1	1	-
IS_749	750	.	.	.	.	chr4	123208060	123208136	1	1	+
IS_750	751	.	.	.	.	chr4	127505811	127505887	1	1	-
IS_751	752	.	.	.	.	chr4	136254248	136254324	1	1	+
IS_752	753	.	.	.	.	chr4	141791764	141791840	1	1	+
IS_753	754	.	.	.	.	chr4	149034185	149034234	1	1	+
IS_754	755	.	.	.	.	chr4	149316850	149316926	1	1	-
IS_755	756	.	.	.	.	chr4	155975422	155975498	1	1	-
IS_756	757	.	.	.	.	chr4	164881000	164881076	1	1	+
IS_757	758	.	.	.	.	chr4	184820622	184820674	1	1	+
IS_758	759	.	.	.	.	chr4	188951438	188951514	1	1	+
IS_759		.	.	.	.	chr5	5280040	5280116	1	1	-
IS_760		.	.	.	.	chr5	8465926	8465997	1	1	-
IS_761		.	.	.	.	chr5	9807921	9807997	1	1	+
IS_762		.	.	.	.	chr5	10857049	10857125	1	1	-
IS_763		.	.	.	.	chr5	10870809	10870885	1	1	-

IS_764	.	.	.	.	chr5	12259051	12259127	1	1	-
IS_765	.	.	.	.	chr5	13582743	13582819	1	1	+
IS_766	.	.	.	.	chr5	18648608	18648684	1	1	+
IS_767	.	.	.	.	chr5	21540932	21540998	1	1	-
IS_768	.	.	.	.	chr5	25894510	25894586	1	1	-
IS_769	.	.	.	.	chr5	30474320	30474396	1	1	+
IS_770	.	.	.	.	chr5	33865384	33865460	1	1	+
IS_771	.	.	.	.	chr5	39312474	39312550	1	1	-
IS_772	.	.	.	.	chr5	39626733	39626809	1	1	-
IS_773	.	.	.	.	chr5	41133203	41133279	1	1	+
IS_774	.	.	.	.	chr5	42914861	42914937	1	1	+
IS_775	.	.	.	.	chr5	44981660	44981730	1	1	-
IS_776	.	.	.	.	chr5	83718711	83718787	1	1	+
IS_777	.	.	.	.	chr5	88065680	88065756	1	1	+
IS_778	.	.	.	.	chr5	89576421	89576497	1	1	-
IS_779	.	.	.	.	chr5	96285358	96285434	1	1	-
IS_780	.	.	.	.	chr5	99050648	99050724	1	1	+
IS_781	.	.	.	.	chr5	104116653	104116729	1	1	-
IS_782	.	.	.	.	chr5	122990866	122990942	1	1	+
IS_783	.	.	.	.	chr5	135703793	135703869	1	1	-
IS_784	.	.	.	.	chr5	140692808	140692855	1	1	+
IS_785	.	.	.	.	chr5	143165684	143165760	1	1	-
IS_786	.	.	.	.	chr5	173132539	173132615	1	1	-
IS_787	.	.	.	.	chr5	176051252	176051328	1	1	+
IS_788	.	.	.	.	chr6	2551318	2551394	1	1	+
IS_789	.	.	.	.	chr6	4259285	4259323	1	1	-
IS_790	.	.	.	.	chr6	4409803	4409879	1	1	-
IS_791	.	.	.	.	chr6	5942340	5942416	1	1	+
IS_792	.	.	.	.	chr6	8400611	8400655	1	1	+
IS_793	.	.	.	.	chr6	9100843	9100890	1	1	-
IS_794	.	.	.	.	chr6	14789724	14789800	1	1	+
IS_795	.	.	.	.	chr6	16401411	16401487	1	1	-
IS_796	.	.	.	.	chr6	20625020	20625069	1	1	-
IS_797	.	.	.	.	chr6	22686662	22686738	1	1	+
IS_798	.	.	.	.	chr6	36011965	36012041	1	1	-
IS_799	.	.	.	.	chr6	36669428	36669504	1	1	+
IS_800	.	.	.	.	chr6	46521050	46521126	1	1	-
IS_801	.	.	.	.	chr6	47075036	47075112	1	1	-
IS_802	.	.	.	.	chr6	47680082	47680158	1	1	+
IS_803	.	.	.	.	chr6	47979041	47979117	1	1	+
IS_804	.	.	.	.	chr6	58779056	58779107	1	1	-
IS_805	.	.	.	.	chr6	66667624	66667700	1	1	+
IS_806	.	.	.	.	chr6	68619525	68619601	1	1	+
IS_807	.	.	.	.	chr6	71239738	71239814	1	1	+
IS_808	.	.	.	.	chr6	72479754	72479830	1	1	-
IS_809	.	.	.	.	chr6	80931940	80932016	1	1	-
IS_810	.	.	.	.	chr6	85963279	85963320	1	1	+
IS_811	.	.	.	.	chr6	87840640	87840716	1	1	-
IS_812	.	.	.	.	chr6	88265076	88265147	1	1	-
IS_813	.	.	.	.	chr6	90133735	90133811	1	1	+
IS_814	.	.	.	.	chr6	92269288	92269364	1	1	-
IS_815	.	.	.	.	chr6	96583702	96583778	1	1	+
IS_816	.	.	.	.	chr6	99252161	99252237	1	1	-
IS_817	.	.	.	.	chr6	101263629	101263705	1	1	-
IS_818	.	.	.	.	chr6	104221949	104222015	1	1	+
IS_819	.	.	.	.	chr6	105309227	105309303	1	1	+
IS_820	.	.	.	.	chr6	107664767	107664843	1	1	+
IS_821	.	.	.	.	chr6	111250142	111250218	1	1	+
IS_822	.	.	.	.	chr6	142601428	142601504	1	1	+

IS_823	.	.	.	.	chr6	143171606	143171682	1	1	+
IS_824	.	.	.	.	chr6	143984676	143984742	1	1	-
IS_825	.	.	.	.	chr6	146308835	146308911	1	1	+
IS_826	.	.	.	.	chr6	147033757	147033833	1	1	-
IS_827	.	.	.	.	chr6	151833342	151833418	1	1	-
IS_828	.	.	.	.	chr6	155840569	155840645	1	1	-
IS_829	.	.	.	.	chr6	157172922	157172998	1	1	+
IS_830	.	.	.	.	chr6	158830552	158830628	1	1	-
IS_831	.	.	.	.	chr6	167099309	167099385	1	1	+
IS_832	.	.	.	.	chr6	168308030	168308106	1	1	-
IS_833	.	.	.	.	chr6	170054317	170054393	1	1	+
IS_834	.	.	.	.	chr7	8841288	8841364	1	1	-
IS_835	.	.	.	.	chr7	9482943	9483019	1	1	-
IS_836	.	.	.	.	chr7	18799230	18799306	1	1	+
IS_837	.	.	.	.	chr7	19433169	19433214	1	1	-
IS_838	.	.	.	.	chr7	20863949	20864025	1	1	+
IS_839	.	.	.	.	chr7	31695341	31695417	1	1	-
IS_840	.	.	.	.	chr7	31955993	31956069	1	1	-
IS_841	.	.	.	.	chr7	32219921	32219997	1	1	-
IS_842	.	.	.	.	chr7	40167338	40167387	1	1	+
IS_843	.	.	.	.	chr7	40898012	40898077	1	1	+
IS_844	.	.	.	.	chr7	52926134	52926190	1	1	-
IS_845	.	.	.	.	chr7	64133900	64133976	1	1	-
IS_846	.	.	.	.	chr7	80682990	80683066	1	1	+
IS_847	.	.	.	.	chr7	81785115	81785177	1	1	+
IS_848	.	.	.	.	chr7	82450525	82450601	1	1	-
IS_849	.	.	.	.	chr7	82774776	82774852	1	1	-
IS_850	.	.	.	.	chr7	83112916	83112992	1	1	-
IS_851	.	.	.	.	chr7	84273786	84273862	1	1	-
IS_852	.	.	.	.	chr7	86255854	86255930	1	1	+
IS_853	.	.	.	.	chr7	93205596	93205672	1	1	-
IS_854	.	.	.	.	chr7	101696424	101696500	1	1	-
IS_855	.	.	.	.	chr7	103937906	103937964	1	1	-
IS_856	.	.	.	.	chr7	107674219	107674295	1	1	-
IS_857	.	.	.	.	chr7	116924030	116924106	1	1	-
IS_858	.	.	.	.	chr7	125294565	125294641	1	1	-
IS_859	.	.	.	.	chr7	131369314	131369390	1	1	+
IS_860	.	.	.	.	chr7	144727708	144727784	1	1	-
IS_861	.	.	.	.	chr7	150985989	150986065	1	1	-
IS_862	.	.	.	.	chr7	158859033	158859109	1	1	+
IS_863	.	.	.	.	chr8	892281	892357	1	1	-
IS_864	.	.	.	.	chr8	11330735	11330811	1	1	+
IS_865	.	.	.	.	chr8	16509944	16510020	1	1	+
IS_866	.	.	.	.	chr8	18397649	18397725	1	1	-
IS_867	.	.	.	.	chr8	20673558	20673634	1	1	-
IS_868	.	.	.	.	chr8	31391330	31391406	1	1	-
IS_869	.	.	.	.	chr8	33105118	33105166	1	1	-
IS_870	.	.	.	.	chr8	36722996	36723072	1	1	-
IS_871	.	.	.	.	chr8	39539633	39539709	1	1	+
IS_872	.	.	.	.	chr8	40273319	40273395	1	1	+
IS_873	.	.	.	.	chr8	55548307	55548383	1	1	+
IS_874	.	.	.	.	chr8	58335084	58335160	1	1	+
IS_875	.	.	.	.	chr8	67314734	67314810	1	1	-
IS_876	.	.	.	.	chr8	68115933	68116009	1	1	-
IS_877	.	.	.	.	chr8	82386494	82386570	1	1	+
IS_878	.	.	.	.	chr8	84271367	84271443	1	1	-
IS_879	.	.	.	.	chr8	84668728	84668804	1	1	+
IS_880	.	.	.	.	chr8	88459236	88459312	1	1	+
IS_881	.	.	.	.	chr8	89818305	89818381	1	1	+

IS_882	.	.	.	.	chr8	94920535	94920611	1	1	+
IS_883	.	.	.	.	chr8	101900202	101900243	1	1	+
IS_884	.	.	.	.	chr8	102768237	102768313	1	1	-
IS_885	.	.	.	.	chr8	103123719	103123795	1	1	-
IS_886	.	.	.	.	chr8	106011502	106011578	1	1	+
IS_887	.	.	.	.	chr8	111027098	111027174	1	1	-
IS_888	.	.	.	.	chr8	116967491	116967567	1	1	+
IS_889	.	.	.	.	chr8	117089652	117089721	1	1	+
IS_890	.	.	.	.	chr8	118502322	118502398	1	1	+
IS_891	.	.	.	.	chr8	120603310	120603386	1	1	+
IS_892	.	.	.	.	chr8	121709190	121709266	1	1	-
IS_893	.	.	.	.	chr8	134047317	134047368	1	1	-
IS_894	.	.	.	.	chr8	139128187	139128263	1	1	-
IS_895	.	.	.	.	chr8	139527505	139527581	1	1	+
IS_896	.	.	.	.	chr9	10821534	10821610	1	1	-
IS_897	.	.	.	.	chr9	20314162	20314224	1	1	+
IS_898	.	.	.	.	chr9	20923419	20923484	1	1	+
IS_899	.	.	.	.	chr9	27346255	27346331	1	1	+
IS_900	.	.	.	.	chr9	31987317	31987393	1	1	+
IS_901	.	.	.	.	chr9	34069364	34069440	1	1	+
IS_902	.	.	.	.	chr9	34830587	34830663	1	1	+
IS_903	.	.	.	.	chr9	71039807	71039883	1	1	+
IS_904	.	.	.	.	chr9	77132173	77132249	1	1	-
IS_905	.	.	.	.	chr9	84569515	84569591	1	1	-
IS_906	.	.	.	.	chr9	86041708	86041784	1	1	-
IS_907	.	.	.	.	chr9	94645624	94645700	1	1	-
IS_908	.	.	.	.	chr9	104198448	104198506	1	1	-
IS_909	.	.	.	.	chr9	119874202	119874278	1	1	-
IS_910	.	.	.	.	chr9	124200264	124200340	1	1	-
IS_911	.	.	.	.	chr9	134006690	134006766	1	1	-
IS_912	.	.	.	.	chr9	134855917	134855993	1	1	+
IS_913	.	.	.	.	chr9	138728213	138728289	1	1	-
IS_914	.	.	.	.	chr9	140896508	140896584	1	1	+
IS_915	.	.	.	.	chr10	486658	486734	1	1	+
IS_916	.	.	.	.	chr10	6194961	6195037	1	1	-
IS_917	.	.	.	.	chr10	8893304	8893380	1	1	+
IS_918	.	.	.	.	chr10	10304187	10304263	1	1	-
IS_919	.	.	.	.	chr10	14607426	14607502	1	1	+
IS_920	.	.	.	.	chr10	17146958	17147034	1	1	+
IS_921	.	.	.	.	chr10	19465157	19465233	1	1	+
IS_922	.	.	.	.	chr10	23758294	23758370	1	1	+
IS_923	.	.	.	.	chr10	24368917	24368993	1	1	+
IS_924	.	.	.	.	chr10	32662525	32662583	1	1	-
IS_925	.	.	.	.	chr10	34367475	34367551	1	1	+
IS_926	.	.	.	.	chr10	42385144	42385220	1	1	-
IS_927	.	.	.	.	chr10	48345327	48345403	1	1	-
IS_928	.	.	.	.	chr10	62810917	62810993	1	1	-
IS_929	.	.	.	.	chr10	66161360	66161436	1	1	-
IS_930	.	.	.	.	chr10	67120260	67120318	1	1	-
IS_931	.	.	.	.	chr10	67892470	67892546	1	1	+
IS_932	.	.	.	.	chr10	68508520	68508596	1	1	-
IS_933	.	.	.	.	chr10	82258242	82258318	1	1	-
IS_934	.	.	.	.	chr10	87098628	87098704	1	1	+
IS_935	.	.	.	.	chr10	96384279	96384355	1	1	+
IS_936	.	.	.	.	chr10	101215978	101216054	1	1	-
IS_937	.	.	.	.	chr10	114152759	114152835	1	1	+
IS_938	.	.	.	.	chr10	115012326	115012402	1	1	-
IS_939	.	.	.	.	chr10	115425792	115425868	1	1	-
IS_940	.	.	.	.	chr10	115756554	115756607	1	1	-

IS_941	.	.	.	.	chr10	123033234	123033310	1	1	-
IS_942	.	.	.	.	chr10	123040448	123040524	1	1	-
IS_943	.	.	.	.	chr10	125389730	125389806	1	1	-
IS_944	.	.	.	.	chr11	908576	908652	1	1	+
IS_945	.	.	.	.	chr11	6102147	6102223	1	1	-
IS_946	.	.	.	.	chr11	8476600	8476676	1	1	-
IS_947	.	.	.	.	chr11	13190323	13190399	1	1	-
IS_948	.	.	.	.	chr11	14940164	14940240	1	1	-
IS_949	.	.	.	.	chr11	18631000	18631076	1	1	-
IS_950	.	.	.	.	chr11	31137683	31137759	1	1	+
IS_951	.	.	.	.	chr11	33524405	33524481	1	1	+
IS_952	.	.	.	.	chr11	41223939	41224015	1	1	+
IS_953	.	.	.	.	chr11	55955620	55955696	1	1	+
IS_954	.	.	.	.	chr11	65899973	65900049	1	1	-
IS_955	.	.	.	.	chr11	66236825	66236901	1	1	-
IS_956	.	.	.	.	chr11	67099155	67099231	1	1	-
IS_957	.	.	.	.	chr11	69140581	69140657	1	1	+
IS_958	.	.	.	.	chr11	83410576	83410655	1	1	+
IS_959	.	.	.	.	chr11	84475346	84475422	1	1	-
IS_960	.	.	.	.	chr11	84872731	84872782	1	1	-
IS_961	.	.	.	.	chr11	95031529	95031605	1	1	-
IS_962	.	.	.	.	chr11	96879566	96879642	1	1	+
IS_963	.	.	.	.	chr11	108526917	108526993	1	1	-
IS_964	.	.	.	.	chr11	109589825	109589901	1	1	-
IS_965	.	.	.	.	chr11	110159534	110159610	1	1	+
IS_966	.	.	.	.	chr11	111186686	111186754	1	1	+
IS_967	.	.	.	.	chr12	15241052	15241128	1	1	+
IS_968	.	.	.	.	chr12	19007719	19007795	1	1	-
IS_969	.	.	.	.	chr12	30918874	30918947	1	1	-
IS_970	.	.	.	.	chr12	34737336	34737412	1	1	-
IS_971	.	.	.	.	chr12	41870051	41870114	1	1	+
IS_972	.	.	.	.	chr12	45454520	45454596	1	1	-
IS_973	.	.	.	.	chr12	55595151	55595227	1	1	+
IS_974	.	.	.	.	chr12	61469141	61469217	1	1	+
IS_975	.	.	.	.	chr12	64608249	64608325	1	1	+
IS_976	.	.	.	.	chr12	66015130	66015206	1	1	+
IS_977	.	.	.	.	chr12	67759201	67759277	1	1	+
IS_978	.	.	.	.	chr12	68157542	68157618	1	1	-
IS_979	.	.	.	.	chr12	72037370	72037446	1	1	-
IS_980	.	.	.	.	chr12	73357023	73357071	1	1	-
IS_981	.	.	.	.	chr12	75625514	75625590	1	1	-
IS_982	.	.	.	.	chr12	76288976	76289052	1	1	+
IS_983	.	.	.	.	chr12	76857471	76857514	1	1	-
IS_984	.	.	.	.	chr12	77094504	77094580	1	1	-
IS_985	.	.	.	.	chr12	77747892	77747948	1	1	-
IS_986	.	.	.	.	chr12	78280517	78280593	1	1	+
IS_987	.	.	.	.	chr12	87427843	87427890	1	1	+
IS_988	.	.	.	.	chr12	88257400	88257476	1	1	+
IS_989	.	.	.	.	chr12	105111238	105111314	1	1	+
IS_990	.	.	.	.	chr12	108631131	108631194	1	1	-
IS_991	.	.	.	.	chr12	118537110	118537186	1	1	+
IS_992	.	.	.	.	chr12	127433978	127434054	1	1	+
IS_993	.	.	.	.	chr12	129011454	129011514	1	1	-
IS_994	.	.	.	.	chr12	131047356	131047432	1	1	+
IS_995	.	.	.	.	chr13	20560670	20560746	1	1	-
IS_996	.	.	.	.	chr13	21449955	21450031	1	1	-
IS_997	.	.	.	.	chr13	27379680	27379756	1	1	-
IS_998	.	.	.	.	chr13	30496712	30496788	1	1	-
IS_999	.	.	.	.	chr13	35640836	35640889	1	1	-

IS_1000	.	.	.	.	chr13	39899102	39899178	1	1	-
IS_1001	.	.	.	.	chr13	40372650	40372726	1	1	-
IS_1002	.	.	.	.	chr13	47504693	47504769	1	1	-
IS_1003	.	.	.	.	chr13	51234664	51234740	1	1	-
IS_1004	.	.	.	.	chr13	51622457	51622533	1	1	-
IS_1005	.	.	.	.	chr13	58605825	58605901	1	1	-
IS_1006	.	.	.	.	chr13	61223742	61223818	1	1	+
IS_1007	.	.	.	.	chr13	63898827	63898903	1	1	-
IS_1008	.	.	.	.	chr13	64815405	64815481	1	1	-
IS_1009	.	.	.	.	chr13	70250905	70250981	1	1	+
IS_1010	.	.	.	.	chr13	71043484	71043560	1	1	+
IS_1011	.	.	.	.	chr13	71981035	71981111	1	1	+
IS_1012	.	.	.	.	chr13	74693634	74693710	1	1	+
IS_1013	.	.	.	.	chr13	77207887	77207963	1	1	+
IS_1014	.	.	.	.	chr13	80120513	80120589	1	1	-
IS_1015	.	.	.	.	chr13	80770056	80770132	1	1	+
IS_1016	.	.	.	.	chr13	81106086	81106162	1	1	+
IS_1017	.	.	.	.	chr13	81454912	81454988	1	1	+
IS_1018	.	.	.	.	chr13	83797536	83797608	1	1	-
IS_1019	.	.	.	.	chr13	86429756	86429832	1	1	-
IS_1020	.	.	.	.	chr13	93308468	93308524	1	1	+
IS_1021	.	.	.	.	chr13	97694740	97694816	1	1	+
IS_1022	.	.	.	.	chr13	100771230	100771306	1	1	-
IS_1023	.	.	.	.	chr13	105463810	105463886	1	1	+
IS_1024	.	.	.	.	chr14	25435343	25435419	1	1	-
IS_1025	.	.	.	.	chr14	25954515	25954591	1	1	-
IS_1026	.	.	.	.	chr14	26288262	26288338	1	1	+
IS_1027	.	.	.	.	chr14	31317875	31317951	1	1	-
IS_1028	.	.	.	.	chr14	36147024	36147100	1	1	+
IS_1029	.	.	.	.	chr14	37357297	37357373	1	1	+
IS_1030	.	.	.	.	chr14	37455288	37455364	1	1	-
IS_1031	.	.	.	.	chr14	44049938	44050014	1	1	-
IS_1032	.	.	.	.	chr14	50829986	50830062	1	1	+
IS_1033	.	.	.	.	chr14	51068652	51068724	1	1	-
IS_1034	.	.	.	.	chr14	53064808	53064847	1	1	-
IS_1035	.	.	.	.	chr14	54432981	54433057	1	1	-
IS_1036	.	.	.	.	chr14	58121590	58121666	1	1	-
IS_1037	.	.	.	.	chr14	64280058	64280134	1	1	+
IS_1038	.	.	.	.	chr14	64871515	64871584	1	1	-
IS_1039	.	.	.	.	chr14	67712739	67712815	1	1	+
IS_1040	.	.	.	.	chr14	68417902	68417978	1	1	+
IS_1041	.	.	.	.	chr14	69704791	69704867	1	1	+
IS_1042	.	.	.	.	chr14	75994172	75994248	1	1	-
IS_1043	.	.	.	.	chr14	80326751	80326827	1	1	-
IS_1044	.	.	.	.	chr14	86672467	86672530	1	1	-
IS_1045	.	.	.	.	chr14	94281778	94281854	1	1	+
IS_1046	.	.	.	.	chr14	99115454	99115530	1	1	+
IS_1047	.	.	.	.	chr14	104369507	104369583	1	1	-
IS_1048	.	.	.	.	chr15	20609489	20609565	1	1	-
IS_1049	.	.	.	.	chr15	32046074	32046150	1	1	-
IS_1050	.	.	.	.	chr15	35156440	35156516	1	1	-
IS_1051	.	.	.	.	chr15	40489015	40489091	1	1	-
IS_1052	.	.	.	.	chr15	40493217	40493293	1	1	+
IS_1053	.	.	.	.	chr15	42370886	42370962	1	1	-
IS_1054	.	.	.	.	chr15	42421811	42421887	1	1	-
IS_1055	.	.	.	.	chr15	48738059	48738135	1	1	-
IS_1056	.	.	.	.	chr15	62667667	62667743	1	1	-
IS_1057	.	.	.	.	chr15	67452361	67452437	1	1	-
IS_1058	.	.	.	.	chr15	81879724	81879800	1	1	-

IS_1059	.	.	.	.	chr15	89841431	89841507	1	1	-
IS_1060	.	.	.	.	chr15	92097574	92097650	1	1	+
IS_1061	.	.	.	.	chr15	97880765	97880841	1	1	+
IS_1062	.	.	.	.	chr15	101005338	101005414	1	1	-
IS_1063	.	.	.	.	chr16	8904606	8904657	1	1	-
IS_1064	.	.	.	.	chr16	11386301	11386342	1	1	+
IS_1065	.	.	.	.	chr16	14680730	14680806	1	1	-
IS_1066	.	.	.	.	chr16	49509524	49509600	1	1	-
IS_1067	.	.	.	.	chr16	54160436	54160512	1	1	-
IS_1068	.	.	.	.	chr16	55514013	55514055	1	1	-
IS_1069	.	.	.	.	chr16	57260673	57260749	1	1	-
IS_1070	.	.	.	.	chr16	63645614	63645690	1	1	+
IS_1071	.	.	.	.	chr16	88758585	88758661	1	1	-
IS_1072	.	.	.	.	chr17	6016432	6016508	1	1	+
IS_1073	.	.	.	.	chr17	11110079	11110155	1	1	+
IS_1074	.	.	.	.	chr17	14835191	14835257	1	1	-
IS_1075	.	.	.	.	chr17	20937015	20937092	1	1	+
IS_1076	.	.	.	.	chr17	25604403	25604479	1	1	-
IS_1077	.	.	.	.	chr17	33250313	33250389	1	1	+
IS_1078	.	.	.	.	chr17	40567804	40567880	1	1	+
IS_1079	.	.	.	.	chr17	49798596	49798672	1	1	-
IS_1080	.	.	.	.	chr17	52115420	52115496	1	1	-
IS_1081	.	.	.	.	chr17	52954662	52954738	1	1	+
IS_1082	.	.	.	.	chr17	57055481	57055557	1	1	-
IS_1083	.	.	.	.	chr17	57958605	57958660	1	1	-
IS_1084	.	.	.	.	chr17	64055297	64055373	1	1	+
IS_1085	.	.	.	.	chr17	67373007	67373078	1	1	+
IS_1086	.	.	.	.	chr17	70715301	70715377	1	1	-
IS_1087	.	.	.	.	chr18	4507196	4507272	1	1	+
IS_1088	.	.	.	.	chr18	6108862	6108938	1	1	-
IS_1089	.	.	.	.	chr18	9201110	9201186	1	1	+
IS_1090	.	.	.	.	chr18	12237359	12237435	1	1	-
IS_1091	.	.	.	.	chr18	13592638	13592714	1	1	-
IS_1092	.	.	.	.	chr18	18512128	18512176	1	1	+
IS_1093	.	.	.	.	chr18	18518867	18518943	1	1	-
IS_1094	.	.	.	.	chr18	27028673	27028749	1	1	-
IS_1095	.	.	.	.	chr18	27593423	27593499	1	1	+
IS_1096	.	.	.	.	chr18	32140054	32140122	1	1	-
IS_1097	.	.	.	.	chr18	60798071	60798147	1	1	-
IS_1098	.	.	.	.	chr18	62853397	62853473	1	1	-
IS_1099	.	.	.	.	chr18	68652014	68652074	1	1	-
IS_1100	.	.	.	.	chr18	69133525	69133601	1	1	-
IS_1101	.	.	.	.	chr18	74805584	74805660	1	1	-
IS_1102	.	.	.	.	chr18	75003616	75003692	1	1	-
IS_1103	.	.	.	.	chr19	9603678	9603754	1	1	-
IS_1104	.	.	.	.	chr19	11460534	11460610	1	1	-
IS_1105	.	.	.	.	chr19	18123749	18123825	1	1	-
IS_1106	.	.	.	.	chr19	19654388	19654464	1	1	+
IS_1107	.	.	.	.	chr19	23174885	23174961	1	1	-
IS_1108	.	.	.	.	chr19	35240023	35240099	1	1	+
IS_1109	.	.	.	.	chr19	51289091	51289167	1	1	+
IS_1110	.	.	.	.	chr19	55320399	55320475	1	1	-
IS_1111	.	.	.	.	chr19	58020122	58020198	1	1	-
IS_1112	.	.	.	.	chr20	224929	224976	1	1	-
IS_1113	.	.	.	.	chr20	3275640	3275716	1	1	-
IS_1114	.	.	.	.	chr20	32440486	32440562	1	1	+
IS_1115	.	.	.	.	chr20	32898400	32898476	1	1	+
IS_1116	.	.	.	.	chr20	41170079	41170130	1	1	+
IS_1117	.	.	.	.	chr20	42084971	42085047	1	1	+

IS_1118	.	.	.	.	chr20	46178439	46178515	1	1	-
IS_1119	.	.	.	.	chr20	47822716	47822792	1	1	-
IS_1120	.	.	.	.	chr20	51050832	51050908	1	1	+
IS_1121	.	.	.	.	chr20	54048268	54048328	1	1	-
IS_1122	.	.	.	.	chr21	10713304	10713380	1	1	-
IS_1123	.	.	.	.	chr21	17298065	17298141	1	1	+
IS_1124	.	.	.	.	chr21	22480202	22480244	1	1	-
IS_1125	.	.	.	.	chr21	30729250	30729326	1	1	-
IS_1126	.	.	.	.	chr21	32175076	32175152	1	1	+
IS_1127	.	.	.	.	chr21	33781874	33781950	1	1	-
IS_1128	.	.	.	.	chr21	34101192	34101249	1	1	+
IS_1129	.	.	.	.	chr21	39392781	39392857	1	1	-
IS_1130	.	.	.	.	chr21	39787726	39787802	1	1	+
IS_1131	.	.	.	.	chr21	40193497	40193541	1	1	-
IS_1132	.	.	.	.	chr21	42707692	42707768	1	1	-
IS_1133	.	.	.	.	chr21	42822105	42822181	1	1	-
IS_1134	.	.	.	.	chr22	36031180	36031256	1	1	+
IS_1135	.	.	.	.	chr22	38961724	38961800	1	1	+
IS_1136	.	.	.	.	chr22	50480723	50480799	1	1	+
IS_1137	.	.	.	.	chrX	9828475	9828551	1	1	-
IS_1138	.	.	.	.	chrX	11127100	11127176	1	1	+
IS_1139	.	.	.	.	chrX	22909834	22909910	1	1	+
IS_1140	.	.	.	.	chrX	24449350	24449426	1	1	+
IS_1141	.	.	.	.	chrX	37457442	37457518	1	1	-
IS_1142	.	.	.	.	chrX	45777037	45777098	1	1	+
IS_1143	.	.	.	.	chrX	50399951	50400027	1	1	-
IS_1144	.	.	.	.	chrX	52002428	52002504	1	1	+
IS_1145	.	.	.	.	chrX	65812069	65812145	1	1	+
IS_1146	.	.	.	.	chrX	66468575	66468651	1	1	+
IS_1147	.	.	.	.	chrX	75166539	75166615	1	1	-
IS_1148	.	.	.	.	chrX	82444151	82444227	1	1	-
IS_1149	.	.	.	.	chrX	86867352	86867428	1	1	-
IS_1150	.	.	.	.	chrX	89298279	89298355	1	1	-
IS_1151	.	.	.	.	chrX	89826604	89826680	1	1	+
IS_1152	.	.	.	.	chrX	101197678	101197729	1	1	+
IS_1153	.	.	.	.	chrX	102956819	102956895	1	1	-
IS_1154	.	.	.	.	chrX	103074077	103074153	1	1	-
IS_1155	.	.	.	.	chrX	103785516	103785592	1	1	+
IS_1156	.	.	.	.	chrX	105124426	105124502	1	1	-
IS_1157	.	.	.	.	chrX	112027086	112027162	1	1	-
IS_1158	.	.	.	.	chrX	115953221	115953267	1	1	-
IS_1159	.	.	.	.	chrM	15707	15783	1	1	-
IS_1160	.	.	.	.	chrM	16391	16467	1	1	-

Table S2. Basic information of next generation sequencing for each samples

	Fig	Reads			Enrichment
		Total	HIV-1	% of total	
**ACH-2**	1B	1,581,497	3	0.0002	(-)
**ACH-2**	1B	561,948	28,386	5.05	(+)
**J1.1**	1C	98,654	10,998	11.15	(+)
**J-Lat 9.2**	1D	343,686	15,524	4.52	(+)
**J-Lat 10.6**	1D	673,161	26,778	3.98	(+)
**THP-1_NanoLuc #95**	3A	429,242	2,202	0.51	(+)
**THP-1_NanoLuc #225**	3A	429,126	1,879	0.44	(+)
**Jurkat/NL4-3**	4A	460,843	34,678	7.52	(+)

Table S3.

probe name	seequence	length
NL4-3_(M19921)_1	AGGGAGAAGTATTAGTGTGGAAGTTTGACAGCCTCCTAGCATTTCGTCACATGGCCCGAGAGCTGCATCCGGAGTACTACAAAGACTGCTGACATCGAGCTTTCTACAAGGGACTTTCCG	120
NL4-3_(M19921)_2	CTGGGGACTTTCCAGGGAGGTGTGGCCTGGGCGGGACTGGGGAGTGGCGAGCCCTCAGATGCTACATATAAGCAGCTGCTTTTTGCCTGTACTGGGTCTCTCTGGTTAGACCAGATCTGA	120
NL4-3_(M19921)_3	AGTAATTCCAGCAGAGACAGGGCAAGAAACAGCATACTTCCTCTTAAAATTAGCAGGAAGATGGCCAGTAAAAACAGTACATACAGACAATGGCAGCAATTTCACCAGTACTACAGTTAA	120
NL4-3_(M19921)_4	CCAAGTTTGTTTCATGACAAAAGCCTTAGGCATCTCCTATGGCAGGAAGAAGCGGAGACAGCGACGAAGAGCTCATCAGAACAGTCAGACTCATCAAGCTTCTCTATCAAAGCAGTAAGT	120
NL4-3_(M19921)_5	TAGTACATGTAATGCAACCTATAATAGTAGCAATAGTAGCATTAGTAGTAGCAATAATAATAGCAATAGTTGTGTGGTCCATAGTAATCATAGAATATAGGAAAATATTAAGACAAAGAA	120
NL4-3_(M19921)_6	ATAGACAGGTTAATTGATAGACTAATAGAAAGAGCAGAAGACAGTGGCAATGAGAGTGAAGGAGAAGTATCAGCACTTGTGGAGATGGGGGTGGAAATGGGGCACCATGCTCCTTGGGAT	120
NL4-3_(M19921)_7	AGATGCATGAGGATATAATCAGTTTATGGGATCAAAGCCTAAAGCCATGTGTAAAATTAACCCCACTCTGTGTTAGTTTAAAGTGCACTGATTTGAAGAATGATACTAATACCAATAGTA	120
NL4-3_(M19921)_8	TAGCGGGAGAATGATAATGGAGAAAGGAGAGATAAAAAACTGCTCTTTCAATATCAGCACAAGCATAAGAGATAAGGTGCAGAAAGAATATGCATTCTTTTATAAACTTGATATAGTACC	120
NL4-3_(M19921)_9	AATAGATAATACCAGCTATAGGTTGATAAGTTGTAACACCTCAGTCATTACACAGGCCTGTCCAAAGGTATCCTTTGAGCCAATTCCCATACATTATTGTGCCCCGGCTGGTTTTGCGAT	120
NL4-3_(M19921)_10	CCCAACAACAATACAAGAAAAAGTATCCGTATCCAGAGGGGACCAGGGAGAGCATTTGTTACAATAGGAAAAATAGGAAATATGAGACAAGCACATTGTAACATTAGTAGAGCAAAATGG	120
NL4-3_(M19921)_11	ATGCCACTTTAAAACAGATAGCTAGCAAATTAAGAGAACAATTTGGAAATAATAAAACAATAATCTTTAAGCAATCCTCAGGAGGGGACCCAGAAATTGTAACGCACAGTTTTAATTGTG	120
NL4-3_(M19921)_12	TCTACTGTAATTCAACACAACTGTTTAATAGTACTTGGTTTAATAGTACTTGGAGTACTGAAGGGTCAAATAACACTGAAGGAAGTGACACAATCACACTCCCATGCAGAATAAAACAAT	120
NL4-3_(M19921)_13	ATAACAACAATGGGTCCGAGATCTTCAGACCTGGAGGAGGCGATATGAGGGACAATTGGAGAAGTGAATTATATAAATATAAAGTAGTAAAAATTGAACCATTAGGAGTAGCACCCACC	119
NL4-3_(M19921)_14	CTCTGGAACAGATTTGGAATAACATGACCTGGATGGAGTGGGACAGAGAAATTAACAATTACACAAGCTTAATACACTCCTTAATTGAAGAATCGCAAAACCAGCAAGAAAAGAATGAAC	120
NL4-3_(M19921)_15	TGCCACAGCCATAGCAGTAGCTGAGGGGACAGATAGGGTTATAGAAGTATTACAAGCAGCTTATAGAGCTATTCGCCACATACCTAGAAGAATAAGACAGGGCTTGGAAAGGATTTTGCT	120
NL4-3_(M19921)_16	ATGGGTGGCAAGTGGTCAAAAAGTAGTGTGATTGGATGGCCTGCTGTAAGGGAAAGAATGAGACGAGCTGAGCCAGCAGCAGATGGGGTGGGAGCAGTATCTCGAGACCTAGAAAAACAT	120
NL4-3_(M19921)_17	AGCAATCACAAGTAGCAATACAGCAGCTAACAATGCTGCTTGTGCCTGGCTAGAAGCACAAGAGGAGGAAGAGGTGGGTTTTCCAGTCACACCTCAGGTACCTTTAAGACCAATGACTTA	120
NL4-3_(M19921)_18	CTGATTGGCAGAACTACACACCAGGGCCAGGGGTCAGATATCCACTGACCTTTGGATGGTGCTACAAGCTAGTACCAGTTGAGCCAGATAAGGTAGAAGAGGCCAATAAAGGAGAGAACA	120
NL4-3_(M19921)_19	TGACCTTTGGATGGTGCTACAAGCTAGTACCAGTTGAGCCAGATAAGGTAGAAGAGGCCAATAAAGGAGAGAACACCAGCTTGTTACACCCTGTGAGCCTGCATGGAATGGATGACCC	118

Table S4.

Name	5'-3'
ACH2_#3 Left	CTTGCTGAACAAGCAAACCA
ACH2_#3 Right	TGTGTGCCCGTCTGTTGTAT
ACH2_#5 Left	CCCACGTCATAGCATGTGTC
ACH2_#5 Right	TGTGTGCCCGTCTGTTGTAT
Alb F	TGC ATG AGA AAA CGC CAG TAA
Alb R	ATG GTC GCC TGT TCA CCA A
Alb probe	/HEX/TGA CAG AGT / ZEN/ CAC CAA ATG CTG CAC AGA A/3IABkFQ/
795Gag-F (ACH2)	GGTGCGAGAGCGTCAGTATTAAG
795Gag-F (NL4-3)	GGTGCGAGAGCGTCGGTATTAAG
911Gag-R	AGC TCC CTG CTT GCC CAT A
Gag probe	/56-FAM/ AAA ATT CGG /ZEN/ TTA AGG CCA GGA GGA AAG AA/ 3IABkFQ
